# A novel lncRNA DFRV plays a dual function in influenza A virus infection

**DOI:** 10.3389/fmicb.2023.1171423

**Published:** 2023-05-25

**Authors:** Keyu Wang, Meiliang Gong, Sumin Zhao, Chengcai Lai, Lingna Zhao, Sijie Cheng, Min Xia, Yuru Li, Kun Wang, Heqiang Sun, Pingjun Zhu, Yu Zhou, Qiangguo Ao, Xinli Deng

**Affiliations:** ^1^Department of Clinical Laboratory, National Clinical Research Center for Geriatric Diseases, The Second Medical Center of Chinese PLA General Hospital, Beijing, China; ^2^The PLA Rocket Force Characteristic Medical Center, Beijing, China; ^3^Department of Pharmaceutical Sciences, Beijing Institute of Radiation Medicine, Beijing, China; ^4^Beijing Key Laboratory for Immunological Research on Chronic Diseases, School of Medicine and Institute for Immunology, Tsinghua University, Beijing, China; ^5^Center for Disease Prevention and Control, Changde, Hunan, China; ^6^Department of Vascular Cell Biology, Max Plank Institute for Molecular Biomedicine, Münster, Germany; ^7^Department of Respiratory and Critical Care Medicine, National Clinical Research Center for Geriatric Diseases, The Second Medical Center of Chinese PLA General Hospital, Beijing, China; ^8^Department of Nephrology, National Clinical Research Center for Geriatric Diseases, The Second Medical Center of Chinese PLA General Hospital, Beijing, China

**Keywords:** long noncoding RNA, influenza A virus, innate immune, proinflammatory signaling, viral replication

## Abstract

Long noncoding RNAs (lncRNAs) have been associated with a variety of biological activities, including immune responses. However, the function of lncRNAs in antiviral innate immune responses are not fully understood. Here, we identified a novel lncRNA, termed dual function regulating influenza virus (DFRV), elevating in a dose- and time-dependent manner during influenza A virus (IAV) infection, which was dependent on the NFκB signaling pathway. Meanwhile, DFRV was spliced into two transcripts post IAV infection, in which DFRV long suppress the viral replication while DFRV short plays the opposite role. Moreover, DFRV regulates IL-1β and TNF-α via activating several pro-inflammatory signaling cascades, including NFκB, STAT3, PI3K, AKT, ERK1/2 and p38. Besides, DFRV short can inhibit DFRV long expression in a dose-dependent manner. Collectively, our studies reveal that DFRV may act as a potential dual-regulator to preserve innate immune homeostasis in IAV infection.

## Introduction

Influenza is an infectious respiratory disease caused by influenza A and influenza B viruses in humans (Uyeki et al., 2022). Approximately 1 billion infections, 3–5 million cases of serious illness, and 300,000–500,000 deaths are predicted to emerge from annual influenza epidemics, according to the WHO (Krammer et al., 2018). Host innate immunity is a critical barrier that prevents virus reproduction and transmission in new hosts. Both type I interferons [namely, interferon-α (IFNα) and IFNβ) and type III interferons (namely, IFNλ) are essential for the antiviral immune response in epithelial cells (Crotta et al., 2013; Wang et al., 2017). Additionally, several innate immune cells like neutrophils, macrophages, and dendritic cells become activated and secrete pro-inflammatory cytokines (Helft et al., 2012; Tecchio et al., 2014; Pernet et al., 2019), which attracts other innate cells like natural killer cells and pro-inflammatory monocytes to the lung where they can eliminate infected cells and prevent viral infection (Chen et al., 2018; Krammer et al., 2018). Despite the fact that these host reactions to infection are essential for the final viral clearance and activation of adaptive immune responses, while excessive inflammation exacerbates the immunopathology, which is known as “cytokine storm” (Liu, Q. et al., 2016). Although the signaling pathway of antiviral responses has been well characterized, further research is still needed to elucidate the exact mechanism.

Long noncoding RNAs (lncRNAs) are defined as transcripts of more than 200 nucleotides that are not translated into proteins (Kopp and Mendell, 2018; Bridges et al., 2021), and are involved with a variety of biological processes, including cell cycle (Kitagawa et al., 2013), differentiation (Fatica and Bozzoni, 2014; Delas et al., 2017), and metabolism (Sirey et al., 2019), as well as a number of disease (Batista and Chang, 2013; Wang et al., 2013; Huarte, 2015; Li et al., 2021). Recent research indicates that lncRNAs are crucial to the antiviral immune response. For instance, LncRNA PCNAP1 increases tumor growth in hepatocellular carcinoma and affects the replication of the hepatitis B virus (Feng et al., 2019). In vesicular stomatitis virus (VSV) infection, Lnc-Lsm3b effectively competes with viral RNA for binding RIG-I via multibranch loop topologies (Jiang et al., 2018), and Lnczc3h7a promotes a TRIM25-mediated RIG-I antiviral innate immune response (Lin et al., 2019). In influenza virus infection, lncRNA-NRAV promotes IAV replication and pathogenicity by regulating the initial transcription of multiple critical interferon-stimulated genes (ISGs; Ouyang et al., 2014), whereas lncRNA-NEAT1 promotes the expression of antiviral genes such as interleukin-8 (IL-8; Imamura et al., 2014). In a prior study, we identified two lncRNAs called AVAN and IVRPIE, of which AVAN promotes antiviral innate immunity by interacting with TRIM25 and enhancing the transcription of FOXO3a (Lai et al., 2021), while IVRPIE inhibits IAV replication by promoting the expression of IFN and ISGs (Zhao et al., 2020). However, the biological significance and the underlying mechanisms for the lncRNAs in IAV induced immunity and inflammation remain to be further elaborated.

In this study, we defined a novel lncRNA, named DFRV (dual function regulating influenza virus), which plays a dual function in IAV infection. DFRV was dramatically increased in a time-and dose-dependent manner post IAV infection. In addition, DFRV were mainly located in the nucleus, and the rapid amplification of cDNA ends (RACE) data revealed that DFRV has two transcript variants, which showed the opposite function. Moreover, DFRV-long inhibit IAV replication via promoting pro-inflammation signaling pathway, while DFRV-short suppress DFRV-long expression. These results reveal DFRV as a potential dual-regulator to maintain innate immune homeostasis following IAV infection.

## Materials and methods

### Virus

The Academy of Military Medical Sciences provided all of the viruses utilized in this investigation. The Influenza A virus A/Beijing/501/2009 (BJ501, H1N1), A/Wisconsin/67/2005(H3N2), A/Anhui/01/2005 (H5N1), A/Hebei/01/2013 (H7N9), B/Ann Arbor/1/66 (IBV) and Sendai virus (SeV) were propagated in embryonated chicken eggs via the allantoic route. Adenovirus was propagated in Vero cells.

### Cell culture

MDCK, A549 and THP-1 cells were purchased from ATCC; MDCK cells were cultured in DMEM (Gibco) supplemented with 10% FBS and 100 U/mL penicillin–streptomycin; A549 cells were cultured in DMEM/F-12(1:1) basic culture medium (Gibco) supplemented with 10% FBS and 100 U/mL penicillin–streptomycin; and THP-1 cells were cultured in RPMI-1640 medium (Gibco) supplemented with 10% FBS and 100 U/mL penicillin–streptomycin. All of the cells were cultured at 37°C in a humidified atmosphere containing 5% CO_2_.

### Quantitative real-time PCR analysis

Total RNA was extracted from cultured cells using TRIzol reagent (Invitrogen). The commercial PrimeScript RT Master Mix (Takara) was used to create cDNA by reverse transcription, which was then subjected to quantitative PCR on the ABI 7500 PCR System (Applied Biosystems) utilizing TB Green^®^ Premix Ex TaqTM II (Tli RNaseH Plus; Takara). qRT-PCR was performed with primer annealing at 50°C for 2 min, an initial denaturation for 10 min at 95°C, followed by 40 cycles of 15 s at 95°C and 30 s at 60°C and 30 s at 72°C. Primer pairs (Supplementary Table S1) were designed using Primer Premier Software 5.0 and synthesized by Invitrogen. To make primer efficiency curves, the simple cDNA was diluted for a 5-fold gradient, and 2 μL of each diluted-simple was used as the template to amplify the cDNA with the primers of target gene and internal reference gene respectively, and the melting curve was analyzed at 60–95°C. The results showed that the *R*^2^ of each standard curve is 0.99 (example for DFRV primer), and the amplification efficiency of PCR is calculated to be between 90 and 110% (Supplementary Figures 1A,B). The 2^−ΔΔCt^ method was used to calculate expression relative to the internal control, which normalized to GAPDH expression. ABI 7500 SDS software version 1.3 was used to evaluate the quantitative results.

### Plasmid construction and transfection

The DFRV gene was cloned into the pcDNA 3.1(+) plasmid. DFRV-specific Antisense oligonucleotides (ASOs), and negative control ASO were synthesized by Integrated DNA Technologies (IDT). The targeting sequences of the ASOs were as follows: ASO Negative Control: 5′-mG*mC*mG*mA*mC*T*A*T*A*C*G*C*G*C*A*mA*mU*mA*mU*mG-3′, ASO1: 5′-mC*mG*mU*mC*mG*T*C*C*A*A*G*C*C*T*T*mC*mC*mC*mC*mA-3′, ASO2: 5′-mU*mU*mG*mA*mC*A*G*C*G*T*A*G*G*C*A*mC*mC*mA*mG*mC-3′, ASO3: 5′-mA*mC*mU*mU*mG*C*C*A*C*G*T*T*A*G*A*mG*mG*mU*mG*mA-3′. In the above ASO targeting sequences, * represents phosphorothioate bonds and m represents 2′OMe (2′-O-methyl base). Cells were transfected with plasmid or ASOs using jetPRIME (Polyplus) according to the manufacturer’s instructions.

### Bioinformatics analysis of non-coding potential and secondary structure prediction

Coding potential calculator 2 was used to examine the non-coding potential of DFRV^1^ (Kang et al., 2017), and s-fold^2^ (Rennie et al., 2019), MXfold2^3^ (Sato et al., 2021), pKiss^4^ (Janssen and Giegerich, 2015), RNAshapes^5^ (Janssen and Giegerich, 2015), RNAfold^6^ (Dimonte et al., 2021) was used to predict the secondary structure for DFRV.

### Subcellular fractionation

The manufacturer’s recommendations were followed while separating the cytoplasmic and nuclear fractions using the PARIS Kit Protein and RNA Isolation System (Invitrogen). Briefly, Cell Fractionation Buffer was used to lyse A549 cells on ice for 5–10 min. The lysates were centrifuged 1–5 min at 4°C and 500 × *g*. A fresh tube was filled with the supernatant cytoplasmic fraction after it had been aspirated from the nuclear pellet. The nuclear pellets were washed Cell Fractionation Buffer and then vigorously lysed with Cell Disruption Buffer using a pipet or vortex. RNA was extracted using TRIzol Reagent and quantitative PCR was performed to detect DFRV expression in cytoplasmic and nuclear fraction.

### 5′ and 3′ rapid amplification of cDNA ends (RACE)

According to the manufacturer’s instructions, the SMARTerTM RACE cDNA Amplification Kit (Clontech) was used to conduct the 5′ and 3′ RACE analyses. The RACE PCR products were cloned into pMD-19 T vector (Takara) and sequenced. The primers for DFRV RACE were as follows:

5′RACE outer: 5′-GACAGCGTAGGCACCAGCGAGAC-3′,
5′RACE inner: 5′-CTGCTAGCAAGCGACGAATTGCG-3′,
3′RACE outer: 5′-GCGCCAGTTCTTCCTAAAAGGAAAG-3′,
3′RACE inner: 5′-CAATGGTCACCTCTAACGTGGCAAG-3′.

### ELISA

Cytokines levels were measured using an ELISA kit according to the manufacturer’s instructions. Human precoated IL-1β ELISA kit (Cat. NO. 1110122), Human precoated TNF-α ELISA kit (Cat. NO. 1117202) and human IFN-α ELISA kit (Cat. NO. 1110012) were purchased from Dakewe, and human IFN-β ELISA kit (Cat. NO. E-EL-H0085c) were purchased from Elabscience.

### Reagents and antibodies

The primary antibodies used in the analysis were anti-NFκB p65, anti-phospho-NFκB p65 (Ser536), anti-STAT3, anti-phospho-STAT3 (Tyr705), anti-PI3K, anti-phospho-PI3K p85 (Tyr458)/p55 (Tyr199), anti-Akt, anti-phospho-Akt (Thr308), anti-p38, anti-phospho-p38 (Thr180/Tyr182), anti-SAPK/JNK, anti-phospho-SAPK/JNK (Thr183/Tyr185), anti-Erk1/2, anti-phospho-Erk1/2 (Thr202/Tyr204), anti-β-actin (13E5) and anti-rabbit IgG were purchased from Cell Signaling Technology, Western Chemiluminescent Horseradish Peroxidase Substrate was purchased from Millipore Corporation, Signal pathway inhibitor including Fludarabine (STAT1, 100 μM, CAT.NO. T1038), SCH772984 (MEK1/ERK1/ERK2, 100 μM, CAT.NO. T6066), MCC950 (NLRP3, 10 μM, CAT.NO. T3701), Ruxolitinib phosphate (JAK1/2, 100 μM, CAT.NO. T3043), SB203580 (p38 MAPK, 100 μM, CAT.NO. T1764), Hydroxychloroquine sulfate (TLR7/9, 100 μM, CAT.NO. T0951) and SP600125 (JNK1/2/3, 10 μM, CAT.NO. T3109) were purchased from TargetMol, PDTC (NFκB, 100 μM, CAT.NO. S3633) were purchased from SELLECK. For signal pathway inhibitor administration, A549 cells were pretreated with the inhibitor for 12 h and the supernatants were removed, followed by BJ501 infection for 24 h.

### Western blotting

All of the cells were lysed on ice in RIPA buffer (Solarbio) supplemented with protease and phosphatase inhibitor cocktail (100 ×; Thermo Fisher) for 10 min. A quarter volume of 5 × loading dye was added to the supernatants. Then the mixtures were heated at 95°C and stored at − 80°C. SDS-PAGE was used to separate the samples, and they were then transferred to a nitrocellulose filter membrane (Roche). The membranes were blocked for 1 h while shaking at room temperature with 5% non-fat milk (Becton Dickinson) in 1 × Tris-buffered saline and 0.1% Triton 100. The membranes were incubated with primary antibodies followed by horseradish peroxidase-conjugated secondary antibodies. The band was visualized using the Automatic chemiluminescence imaging system (Tanon). The band intensity was analyzed using Quantity One software.

### RNA fluorescence *in situ* hybridization

RNA fluorescence *in situ* hybridization (RNA FISH) was performed as described previously (Liu, L. et al., 2016). Hybridization was carried out using DNA probe sets (Biosearch Technologies) according to the protocol provided by Biosearch Technologies. Cells were observed on a FV1000 confocal laser microscope (Olympus).

### Statistical analyses

GraphPad Prism version 5.0 (GraphPad Software Inc.) was used to conduct all of the statistical analyses. An ANOVA was used to examine the data for measurements taken at a single time point, and a two-tailed t-test was used to further investigate the findings if a significant difference between the groups was found. Statistically significant was defined as *p* < 0.05. All experiments were performed in triplicate.

## Results

### Long noncoding RNA DFRV is significantly up-regulated post IAV infection dependent on NFκB signaling

**Figure 1:**
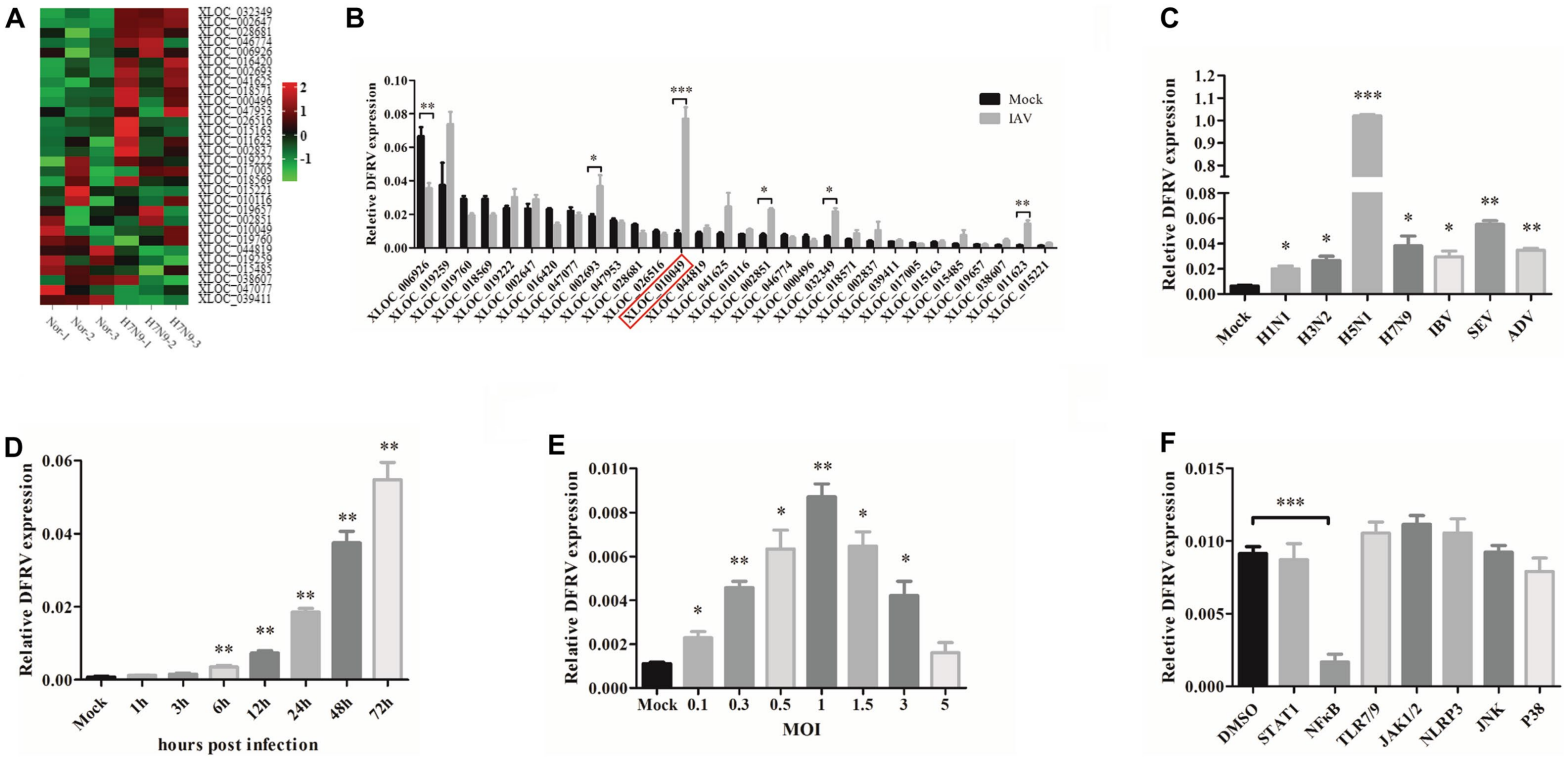
Long noncoding RNA dual function regulating influenza virus (lncRNA DFRV) is significantly elevated in influenza A virus (IAV) infection. **(A)** Cluster heat map showing 30 selected lncRNAs differentially expressed in IAV-infected patient compared with recovery-stage samples based on RNA-seq data (FC > 2; *p* < 0.05). **(B)** The differential expressions of 30 selected lncRNAs were confirmed by RT-PCR in A549 cells stimulated with BJ501 (MOI = 1) or mock for 24 h. DFRV (XLOC_010049) is indicated by red rectangle. **(C)** A549 cells were infected with H1N1, H3N2, H5N1, H7N9, influenza B virus (IBV), Sendai virus (SEV) and Adenovirus (ADV) at MOI = 1 for 24 h, qRT-PCR was performed to determine the DFRV expression. **(D)** A549 cells were infected with BJ501 at different multiplicity of infection (MOI) for 24 h, and **(E)** A549 cells were infected with BJ501 (MOI = 1) for different hours post infection, qRT-PCR was performed to determine the DFRV expression. **(F)** A549 cells were pretreated with 7 different signaling pathway inhibitors for 12 h followed by BJ501 infection for 24 h. qRT-qPCR was performed to determine the DFRV expression. All of the data are shown as the mean ± SD; *n* = 3. ^*^p < 0.05; ^**^p < 0.01;^***^p < 0.001.

To investigate the functions of host lncRNAs during IAV infection, we analyzed whole transcriptional alterations using RNASeq from patients infected with IAV in the acute stage and their matching recovery-stage (9 months after recovery) in a previous study (GSE108807; Lai et al., 2021). According to the basal expression level and fold change (fold change > 2), 30 lncRNA were chosen to confirm the various expression in A549 cell by qRT-PCR, in which XLOC 010049 was highly elevated and has a high basal expression (Figures 1A,B). Therefore, XLOC 010049, termed DFRV, was chosen for further function studies.

Additionally, we discovered that DFRV was increased in A549 and THP-1 cells following influenza virus infection with H1N1, H3N2, H5N1, H7N9, influenza B virus (IBV), other RNA virus (Sendai virus, SEV), and DNA virus (Adenovirus; Figure 1C; Supplementary Figure 2A). Meanwhile, DFRV was upregulated in a dose- and time-dependent manner in A549 cell (Figures 1D,E), and similar result was achieved in the THP-1 cell (Supplementary Figures 2B,C), indicating that it principally functioned in immune cells and lung cells. Moreover, signaling pathway inhibitor was administrated to explore the regulating mechanism of DFRV expression, and we found that DFRV can be inhibited in A549 (Figure 1F) and THP-1 cells (Supplementary Figure 2E) by the kinase inhibitor of NF-κB (PDTC), and the dose-dependent effect of PDTC on inhibiting DFRV expression was validated in A549 cells and THP-1 cells (Supplementary Figures 2D,F). These findings suggest that DFRV was upregulated post virus infection, which was regulated by the NFκB signaling pathway.

### Two transcripts of DFRV have different structures and major located in nucleus

**Figure 2:**
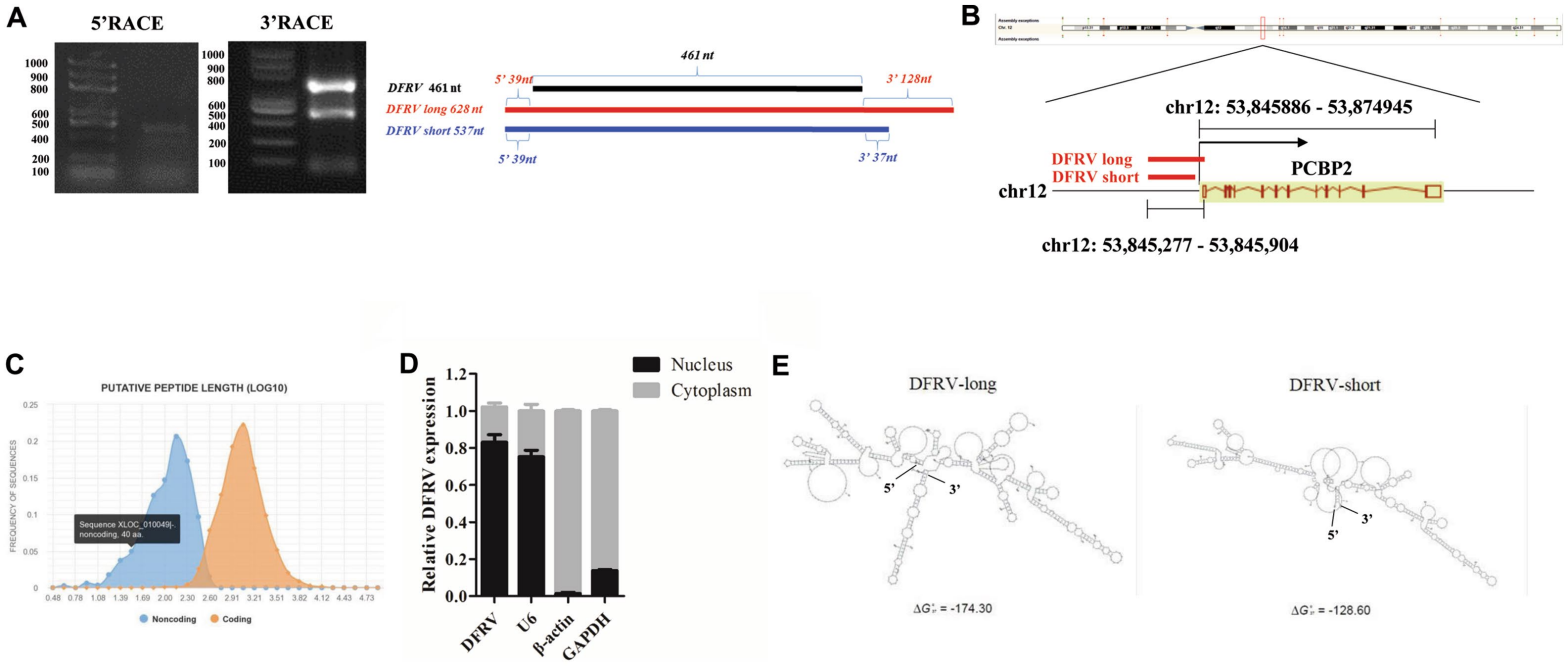
Two transcripts of DFRV have different structures and major located in nucleus. **(A)** The 5′ and 3′ end sequences of DFRV determined by RACE (left) and schematic diagram showing the RACE products of DFRV (right). **(B)** Schematic diagram of gene locus of DFRV. **(C)** Protein-coding potential analysis of DFRV was performed by coding potential calculator 2. **(D)** Cytoplasmic and nuclear fractions from infected A549 cells were separated, and the expression of DFRV were determined by RT-qPCR. U6 RNA served as nuclear gene expression control, β-actin and GAPDH served as cytoplasmic gene control (*n* = 3; means ± SEM). **(E)** Secondary structure predictions of DFRV were performed through S-fold, and the structure with minimum free energy (MEF) of DFRV long (left) and short (right) were exhibited.

To define the basic characters of DFRV, 5′ and 3′ rapid amplification of cDNA ends (RACE) were performed. Interestingly, two transcripts (named DFRV long and DFRV short) was identified. Both of the DFRV transcripts share the same 5′ sequences, while the length of 3′ sequences were 128 and 37 nt respectively, and both of which contain a polyadenylated tail (Figure 2A; Supplementary Table S2). To validate the abundance of two transcripts of DFRV, we designed a pair of qPCR primer in 3′ end of DFRV long to detect the expression of DFRV long (Supplementary Figure 3A). The result showed a similar elevating expression in DFRV total and DFRV long (Supplementary Figure 3B), and the expression level of DFRV long was higher than DFRV short, while the significance was only found in mock groups (Supplementary Figure 3C). The human lncRNA gene DFRV is located on chromosome 12q13.13, overlapping with the sense strand of poly (rC) binding protein 2 (PCBP2) gene (Figure 2B). Meanwhile, DFRV had no protein-coding potential, according to coding potential calculator 2 (Figure 2C). Besides, DFRV is primarily found in the nucleus and is identified by subcellular fractionation (Figure 2D), which is validated by RNA-FISH (Supplementary Figure 3D). Furthermore, the S-fold server was used to estimate the secondary structure of the RNA, and the results revealed that DFRV long had one more stem-loop structure at the 3′ end than DFRV short (Figure 2E), as well as in other four secondary structure prediction software (Supplementary Figures 4A–D). Based on the secondary structure data, we hypothesize that two transcripts of DFRV may play separate roles in IAV infection.

### Dual function regulating influenza virus long suppress the IAV replication *in vitro*

**Figure 3:**
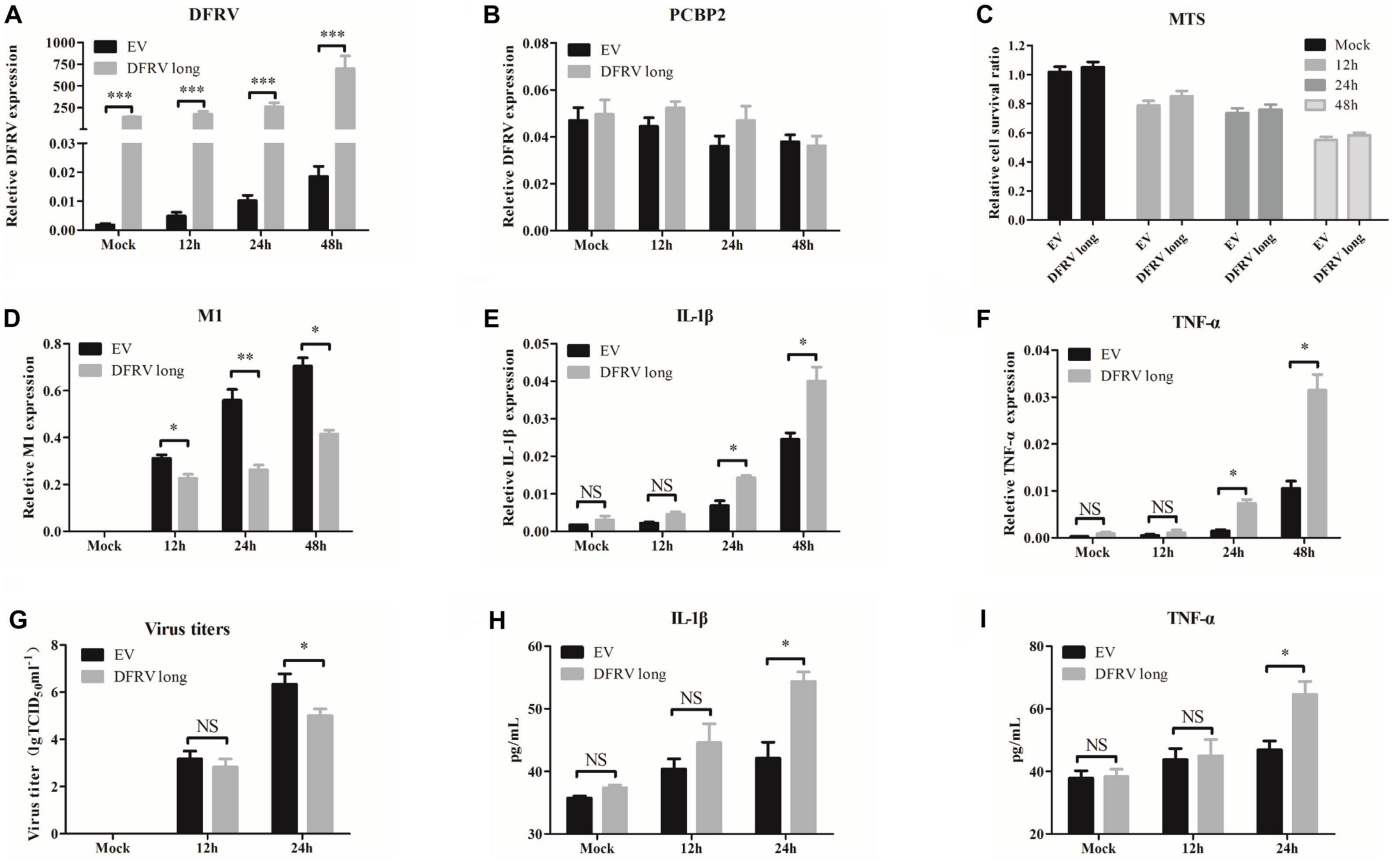
Dual function regulating influenza virus (DFRV) long inhibit viral replication and positively regulate IL-1β and TNF-α in A549 cells. DFRV long were transiently overexpressed in A549 cells for transfected with plasmids for 14 h, and infected with BJ501 (MOI = 1) in 12, 24, and 48 h. The expression level of DFRV **(A)**, PCRP2 **(B)** was determine by qRT-PCR. The cell viability was determined by MTS **(C)**. IAV titers were, respectively, determined by IAV segment M1 expression by qRT-PCR **(D)** and TCID_50_ assay **(G)**. The mRNA expression level of IL-1β **(E)** and TNF-α **(F)** was determined by qRT-PCR. A549 culture supernatants were collected to measure the IL-1β **(H)** and TNF-α **(I)** expression by ELISA. All of the data are shown as the mean ± SD; *n* = 3. ^*^p < 0.05; ^**^p < 0.01;^***^p < 0.001, EV: Empty vector.

To further assess the role of DFRV in IAV infection, A549 cells were transfected with DFRV long plasmid, and the cell viability, virus load, and antiviral related cytokines were measured. The results revealed that DFRV was highly expressed following DFRV long transfection (Figure 3A), while no significant effect was observed in the adjacent gene (PCBP2) by altering DFRV long expression (Figure 3B). Meanwhile, no difference in cell viability was detected using the MTS assay post DFRV long expression (Figure 3C). However, the viral titers were significantly alleviate in DFRV long overexpressing cells than in control cells by qRT-PCR (Figure 3D), and a similar result was achieved by TCID_50_ assay (Figure 3G). Moreover, both the mRNA level in A549 cells and protein level in cell culture supernatant of the pro-inflammation cytokines, IL-1β and TNF-α, were up-regulated in DFRV long overexpressing cells post IAV infection (Figures 3E,F,H,I), whereas the expression of IFN-α and β were negatively regulated (Supplementary Figures 5A-D). These results imply that DFRV long reduce IAV replication, and that pro-inflammatory cytokines, rather than cell death and IFN production, were responsible for the reduction of virus replication.

### Dual function regulating influenza virus short promote the IAV replication *in vitro*

**Figure 4:**
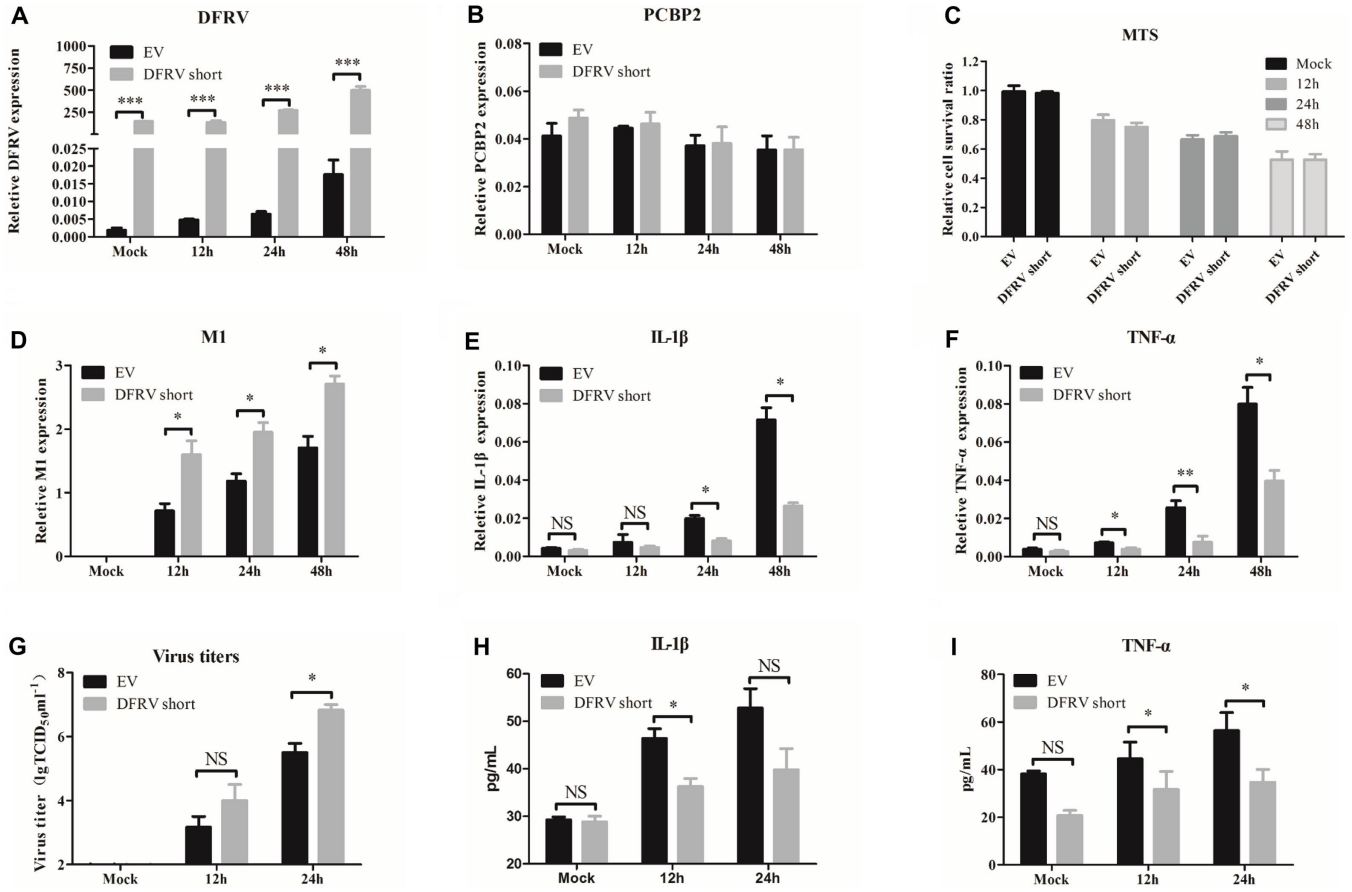
Dual function regulating influenza virus (DFRV) short promote viral replication and negatively regulate IL-1β and TNF-α in A549 cells. DFRV short were transiently overexpressed in A549 cells for transfected with plasmids for 14 h, and infected with BJ501 (MOI = 1) in 12, 24 and 48 h. The expression level of DFRV **(A)**, PCRP2 **(B)** was determine by qRT-PCR. The cell viability was determined by MTS **(C)**. IAV titers were, respectively, determined by IAV segment M1 expression by qRT-PCR **(D)** and TCID_50_ assay **(G)**. The mRNA expression level of IL-1β **(E)** and TNF-α **(F)** was determined by qRT-PCR. A549 culture supernatants were collected to measure the IL-1β **(H)** and TNF-α **(I)** expression by ELISA. All of the data are shown as the mean ± SD; *n* = 3. ^*^p < 0.05; ^**^p < 0.01;^***^p < 0.001, EV: Empty vector.

Consisted with the function in DFRV long, overexpression of DFRV short had no effect on the PCBP2 expression or cell viability (Figures 4A–C). Additionally, contrary to the DFRV long function, DFRV short overexpression exacerbates the virus replication (Figures 4D,G), and both mRNA and protein levels of IL-1β and TNF-α were significantly lower than control cells (Figures 4E,F,H,I). However, overexpression of DFRV short still inhibits the IFN expression as compared to empty vector group (Supplementary Figures 5E–H). Thus, the opposing effects of DFRV short and long in virus replication could be attributed to their distinct impacts on pro-inflammation cytokines.

### Dual function regulating influenza virus long might play a preferential role in inflammatory response

**Figure 5:**
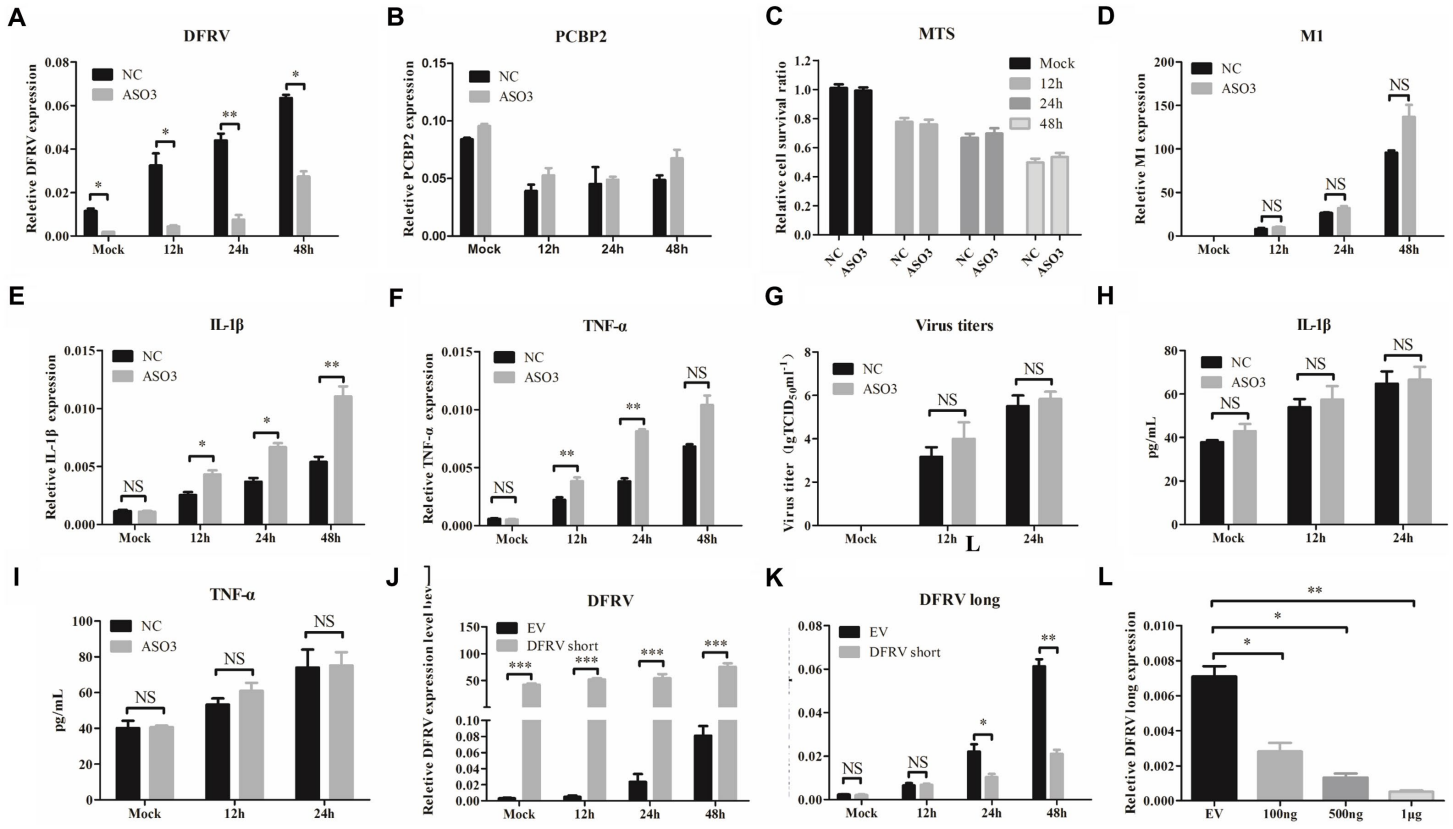
Dual function regulating influenza virus (DFRV) long might play a preferential role in antiviral immune response. DFRV-related ASOs were designed and transfected to A549 cells for 14 h to knockdown the DFRV expression, and infected with BJ501 (MOI = 1) in 12, 24, and 48 h. The expression level of DFRV **(A)**, PCRP2 **(B)** was determine by qRT-PCR. The cell viability was determined by Cell Proliferation Assay (MTS) **(C)**. IAV titers were, respectively, determined by IAV segment M1 expression by qRT-PCR **(D)** and TCID_50_ assay **(G)**. The mRNA expression level of IL-1β **(E)** and TNF-α **(F)** was determined by qRT-PCR. A549 culture supernatants were collected to measure the IL-1β **(H)** and TNF-α **(I)** expression by ELISA. A549 cells were transfected with DFRV short plasmids for 14 h, and infected with BJ501 (MOI = 1) in 12, 24 and 48 h, and DFRV expression level **(J)**, DFRV long expression level **(K)** was determine by qRT-PCR. **(L)** A549 cells were transfected with DFRV short plasmids in different copies for 14 h, and the expression level of DFRV long was determine by qRT-PCR.

To further investigate the function of the two transcripts, we designed 3 DFRV specific Antisense Oligonucleotides (ASO). However, only the last 91 nt in the 3′ end of the two transcripts are different and highly conservative, implying that the target of all 3 ASOs are the common sequence of two transcripts (Supplementary Figure 6A), in which ASO3 achieves the best knockdown efficiency (Supplementary Figure 6B). Following that, A549 cells were transfected with ASO3 to downregulate DFRV expression (Figure 5A), and the effect of DFRV downregulation on IAV viral replication or cytokine expression was examined as previously described. There was no significant difference in PCBP2 expression or cell viability (Figures 5B,C), as well as virus load after two transcripts were knocked down (Figures 5D,G). Contrary to DFRV short overexpression, the mRNA expression level of IL-1β and TNF-α was higher in ASO-transfected cells compared to control cells (Figures 5E,F), while no significance was observed in the protein level of cell culture supernatants (Figures 5H,I). Moreover, ASO3 reversed the downregulation of IFN caused by DFRV overexpression (Supplementary Figure 6C–F). Besides, co-transfection with DFRV long and short showed a satisfactory efficiency in A549 cells (Supplementary Figure 6G), and co-transfection with DFRV long and short had no significant effect on virus load and inflammatory response (Supplementary Figures 6H,I), while IFN-α and -β was significant suppressed post 24 h IAV infection (Supplementary Figures 6 K,L).

To explore the interaction between the two transcripts of DFRV, we determined the expression level of DFRV long in cells transfected with DFRV short (Figure 5J). Interestingly, the results revealed that DFRV short overexpression significantly suppresses DFRV long expression post IAV infection (Figure 5K). In addition, DFRV short significantly inhibit the DFRV long expression in a dose-dependent manner (Figure 5L).

### Dual function regulating influenza virus regulate the inflammatory response via several pro-inflammatory signaling pathways

**Figure 6:**
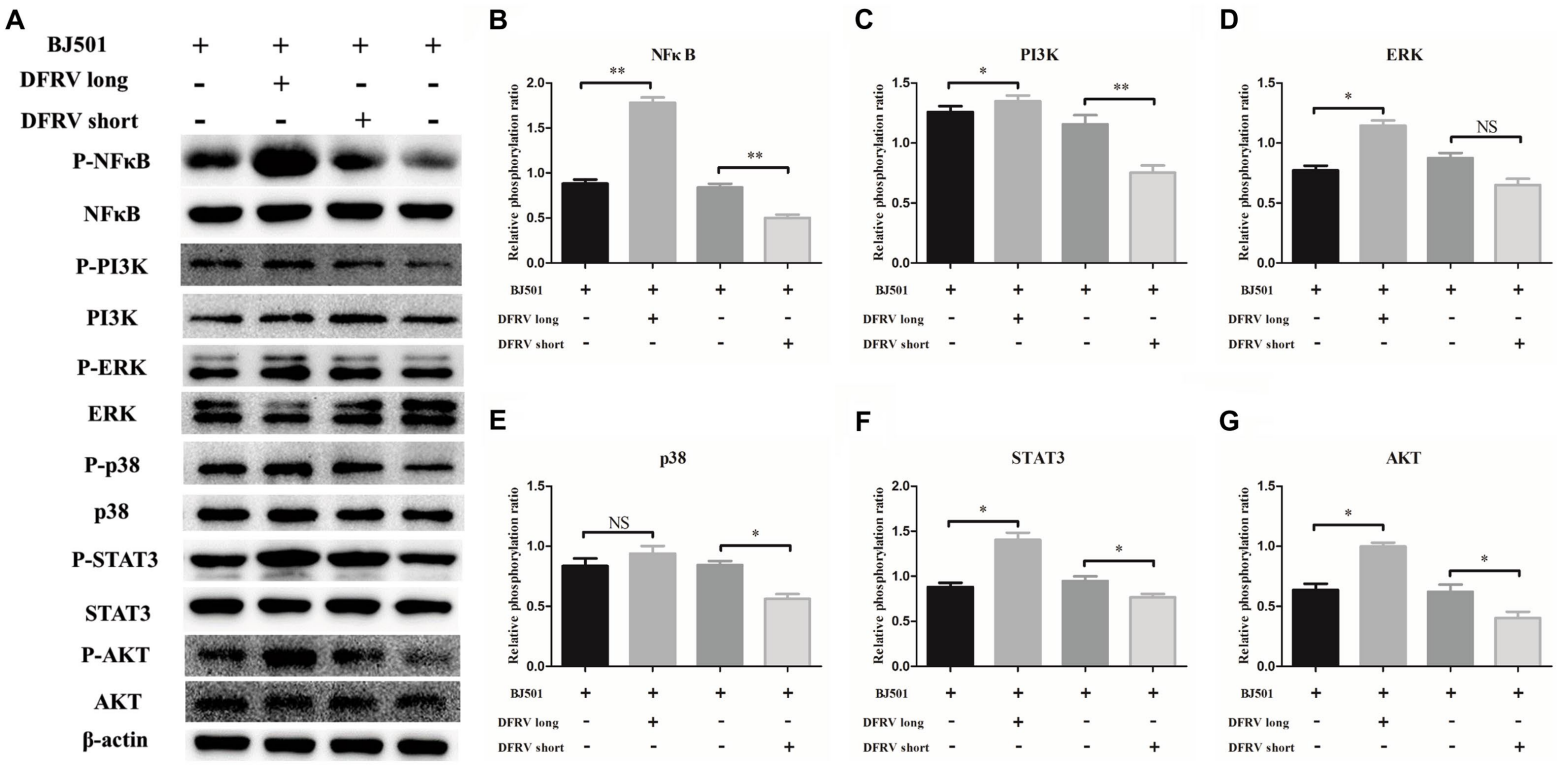
Dual function regulating influenza virus (DFRV) regulate the inflammatory response via several pro-inflammatory signaling pathways. **(A)** Western blot analysis of pro-inflammatory signaling in A549 cells transfected with DFRV long, short and ASO upon BJ501 (MOI = 1) infection for 24 h, β-actin were used as a loading control. **(B-G)** The phosphorylation ratio of NFκB **(B)**, PI3K **(C)**, ERK **(D)**, p38 **(E)**, STAT3 **(F)** and AKT **(G)** was calculated. All of the data are shown as the mean ± SD; *n* = 3. ^*^p < 0.05; ^**^p < 0.01; ^***^p < 0.001, EV: Empty vector.

To elucidate the mechanism of the altered expression in IL-1β and TNF-α induced by DFRV, we examined the pro-inflammatory signaling pathway after DFRV overexpression. As expected, prominent phosphorylation of several pro-inflammatory signaling pathways such as NFκB, MAPKs including ERK1/2 and p38 MAPK, STAT3, PI3K and AKT were observed at 24 h after the DFRV long overexpression, in contrast, these pro-inflammatory signaling phosphorylation were weaker in DFRV short overexpression group, and the altering signaling phosphorylation of ASO were accompanied with pro-inflammatory cytokine expression (Figures 6A–G). Collectively, DFRV long may play a dual role in the antivirals immune response by activating pro-inflammatory signaling pathways.

## Discussion

**Figure 7:**
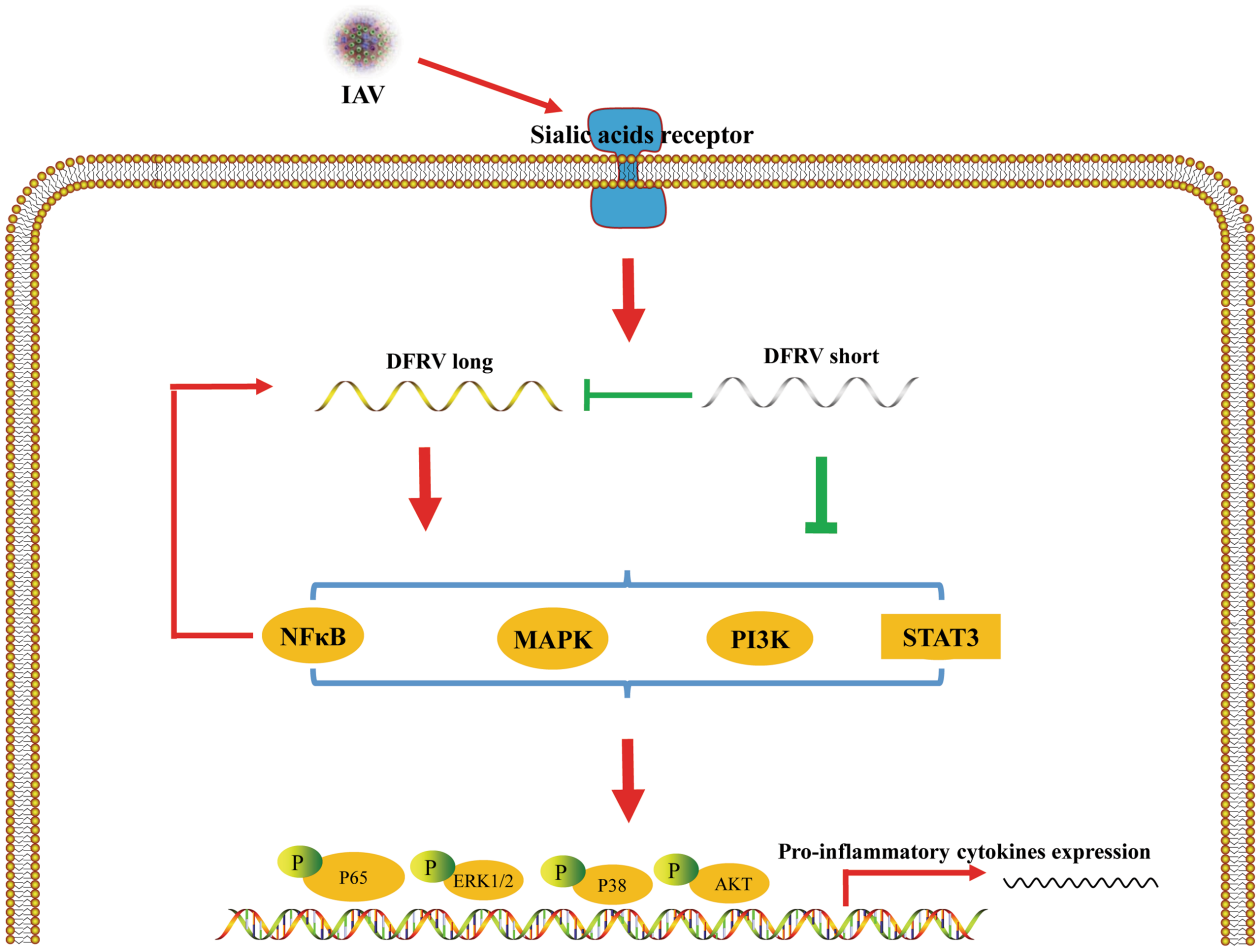
Schematic of the mechanisms by which DFRV regulates antivirus innate immune responses. IAV infection induced DFRV up-regulation, which is regulated by NFκB signaling pathway. DFRV is spliced to DFRV long and short post IAV infection, in which DFRV short inhibit the expression of DFRV long, and DFRV regulate pro-inflammatory cytokines expression via MAPKs, NFκB, STAT3, PI3K-AKT signaling pathways.

LncRNA plays an important role in various biological processes, and emerging research confirms the significance lncRNAs in innate immunity and host–virus interactions (Fortes and Morris, 2016; Basavappa et al., 2019; Liu et al., 2019). It has been demonstrated that a number of lncRNAs regulate the identification by innate immune cells and the transcription of gene expression programs such as NEAT1, MAHAT, OASL-IT1 (Morchikh et al., 2017; Wang et al., 2021; Liu et al., 2022). Here, we discover a novel lncRNA termed DFRV that was significantly up-regulated during IAV infection. DFRV was split into two transcripts, each of which plays an opposing role in antiviral immunity. Moreover, DFRV long suppress viral replication by promoting the expression of IL-1β and TNF-α, and pro-inflammatory signaling pathways including NFκB, STAT3, PI3K-AKT, ERK and p38 were elevated in this biological process. Besides, DFRV long serves as a key component in antivirals immune response, while DFRV short regulates the expression of DFRV long (Figure 7).

It has been extensively investigated how IAV infection cause symptoms in humans. Through the binding of HA to either 2, 3- or 2, 6-linked sialic acids, IAV predominantly targets airway and alveolar epithelial cells (Kobasa et al., 2004; Chutinimitkul et al., 2010; van Riel et al., 2010; Te Velthuis and Fodor, 2016). The innate immune system serves as an effective first line of defense against influenza viruses and is crucial in limiting the anatomic spread of IAV (White et al., 2008; Iwasaki and Pillai, 2014; Wang et al., 2018).Various pattern recognition receptors (PRRs) identify the viral RNA present in infected cells as foreign, activating intracellular immune pathways that result in the release of type I interferons (IFNs), pro-inflammatory cytokines, and chemokines (Durbin et al., 2013; Pothlichet et al., 2013; Iwasaki and Pillai, 2014). In this study, 30 lncRNA were chosen to confirm the differential expression in A549 cell by qRT-PCR, in which XLOC 010049 was highly elevated and has a high basal expression. However, these data were different from RNA-seq. For the RNA-seq, we used neutrophil samples from patients infected with the influenza A (H7N9) virus in the acute stage and their matched recovery-stage samples. Because our primary concern was influenza-induced acute lung injury, differential expression were validated in A549 cells. Inconsistencies in sequencing and validation results may have been exacerbated by differences in sample types (Consortium S M-I, 2014). Simultaneously, discordant information such as the patient’s age and previous disease may lead to contradictory differential expression results (Aird et al., 2011). Meanwhile, we searched the RNA-seq database and found in GSE61517 (Fabozzi et al., 2018), DFRV was significantly increased in BEAS-2B cells 24 h hours post three H3N2 viruses, including two seasonal IAV/H3N2 strains, Brisbane/10/07 and Perth/16/09 (reference strains for past vaccine seasons) and the well-characterized laboratory strain Udorn/307/72, which is consistent with our findings. In our study, DFRV was dramatically up-regulated in A549 and THP-1 cells post IAV infection in a dose- and time-dependent manner, which is dependent on NFκB signaling activation. Besides, the results showed that THP-1 cells were more sensitive to IAV infection than A549 cells as an immune cell, while several studies have shown that IAV replication is more significant in A549 cells than in THP-1 cells (van Riel et al., 2011; Li et al., 2018). Therefore, we chose A549 cells for the following function study.

Furthermore, two transcripts of DFRV were identified by RACE. Both of DFRV long and short were elevated post IAV infection, in which DFRV long was significantly higher than DFRV short without IAV infection, while no significance were found post IAV infection, which indicate a higher up-regulation of DFRV short than DFRV long post IAV infection. Previous research demonstrated that the varied roles of lncRNAs are based on their susceptibility to fold into thermodynamically stable secondary and higher-order structures (Novikova et al., 2012; Mercer and Mattick, 2013). For example, structural alterations in lncRNA H19 influence regulatory functions and serve as optimal targets for further in-depth investigations into their molecular interactions (Martens et al., 2021). Likewise, HOTAIR is composed of four separate structural modules, two of which precisely correspond to predicted protein-binding domains (Somarowthu et al., 2015), and a proper folding structure of the MEG3 RNA molecule is crucial for its biological functions (Zhang et al., 2010). Our findings demonstrated that, in comparison to the DFRV long, the DFRV short lacks a stem-loop structure. Thus, we further explore the role of two transcripts of DFRV. Interestingly, our results showed a diametrically opposed role for DFRV long and short in virus replications.

Because lncRNAs have been shown to affect the expression of neighboring genes, and it has been proposed that many other lncRNAs can also function as local regulators (Engreitz et al., 2016), we investigated the effect of DFRV on the neighboring gene PCBP2 and the result indicated no significant difference, suggesting that the antiviral response of DFRV is independent of PCBP2. Hypercytokinemia, also known as cytokine storm, which is usually present in patients with severe IAV infection and is closely associated to disease severity and may be a predictor of disease progression and mortality (Iwasaki and Medzhitov, 2011; Gui and Chen, 2021). However, cell death and inflammation do not always harm the host; moderate cell death and inflammation can facilitate the elimination of viruses (Kuriakose et al., 2016; Zheng et al., 2020). Hence, we determined whether DFRV had any influence on the activation of cell viability, pro-inflammatory cytokines and IFNs. The results showed there was no significant difference in cell viability levels in A549 cells transfected with DFRV long, short and ASO, while DFRV long positively regulates IL-1β and TNF-α production at both the mRNA and protein levels, whereas DFRV short inhibit those expression. Meanwhile, both DFRV long and short negatively regulate the IFN-α and -β expression. Collectively, these results reveal that DFRV regulate the pro-inflammatory cytokine stimulation in response to IAV replication.

The initiation of an inflammatory response induced by viral infection is dependent on several signaling cascades, including NFκB, JAK–STAT, and MAPKs (Guo et al., 2012; Ding et al., 2017; Li et al., 2020). The NFκB signaling pathway is activated during IAV infection and regulated the expression of important pro-inflammatory cytokines such as IL-1, IL-6, IL-8 and TNF-α (White et al., 2008). STAT3 is a significant transcription factor that is essential for expression of a variety of pro-inflammatory and immune response genes (Pandey et al., 2022; Yao et al., 2022), and a recent study reported that miR-4,485 regulates the production of cytokines by targeting STAT3-PI3K-AKT signaling pathway, thus alleviating H1N1-induced injury in A549 cells (Guo et al., 2020). Furthermore, the dysregulation of pro-inflammatory mediators caused by low-or high-pathogenic influenza has been linked to MAPKs, such as ERK1/2 and p38 MAPK (Kother et al., 2014; Dai et al., 2018; Zhou et al., 2021). To test whether DFRV stimulates these signaling, phosphorylation of NFκB, STAT3, PI3K, AKT, ERK1/2 and p38 was examined by western blotting. As expected, significant difference in the levels of these signaling was observed among the infected DFRV long and short-overexpressing or ASO-transfected cells, which were in consisted with pro-inflammatory cytokine expression. In combination with the major location of DFRV in nucleus, our results further support the idea that DFRV play important roles in antiviral immune response dependent on pro-inflammatory signaling cascades.

It has been acknowledged that the diverse functions of RNA transcripts in human diseases may be interpreted by the distinct structures that result from various splicing modalities (Huang et al., 2018). Previous research has shown that MBNL3 induces the inclusion of exon 4 in the lncRNA-PXN-AS1, and the two major isoforms of lncRNA-PXN-AS1 have distinct effects on Paxillin (PXN; Yuan et al., 2017). LncRNAs and circRNAs are two different types of transcriptional products of the genome, while differential splicing of the same gene can result in the formation of either lncRNAs or circRNAs, even different lncRNAs or circRNAs can be produced from a single gene due to splicing of different exons and introns (Huang et al., 2018). Meanwhile, IAV infection also triggers a widespread program of alternative splicing of host genes, which is a crucial process in the viral life cycle (Thompson and Lynch, 2019; Thompson et al., 2020; Esparza et al., 2022), and a large number of infection-induced splicing processes generate proteins critical for IAV replication (Thompson et al., 2020). Thus, more in depth studies are required to understand the mechanism by triggering alternative splicing to produce two DFRV transcriptions in IAV infection.

However, there are still some limitations in our study. Firstly, because only the last 91 nt in the 3′ end of the two transcripts are different and highly conservative, we cannot design a primer or ASO target for the DFRV short, which limits us to further investigate the interaction between the two transcript of DFRV. Secondly, although mouse genome contains the DFRV homolog sequence, we did not succeed in altering mouse DFRV expression, which remains to be a barrier to *in vivo* studies. Besides, lncRNAs have been reported to regulate gene expression in different ways, including by targeting chromatin modifiers (Herman et al., 2022), acting in cis or in trans (Chen, 2016), interacting with proteins, RNAs, and DNAs (Wu et al., 2017), acting as sponges to sequester RNA-binding proteins (RBPs) and microRNAs (Paraskevopoulou and Hatzigeorgiou, 2016) and so on. Further studies focusing on the interaction factor of DFRV is necessary to elucidate the mechanism of the regulating roles of DFRV in antiviral immunity.

In conclusion, we discovered a novel lncRNA, DFRV, which was elevated in IAV infection and was spliced into two transcripts with the opposite function in viral replication. Moreover, the antiviral functions of DFRV up-regulates IL-1β and TNF-α is dependent on several pro-inflammatory signaling cascades. The precise mechanism of DRFV in antiviral activities needs to be further determined.

## Data availability statement

The datasets presented in this study can be found in online repositories. The names of the repository/repositories and accession number(s) can be found at: https://www.ncbi.nlm.nih.gov/geo/, GSE108807.

## Author contributions

KW, CL, and XD contributed to conception and design of the study. SZ, CL, LZ, and SC performed the experiment. MG, MX, and HS organized the database. KW, MG, LZ, SC, and KW performed the statistical analysis. KW and MG wrote the first draft of the manuscript. PZ, YZ, and QA reviewed and revised the manuscript. YZ, QA, and XD supervised the study. KW and XD take primary responsibility for communication with the journal and editorial office during the submission process, throughout peer review and during publication. All authors contributed to the article and approved the submitted version.

## Funding

This research was funded by “National Natural Science Foundation of China, grant number 82000004 and 81771700” and “Beijing Natural Science Foundation, grant number 7232150.”

## Conflict of interest

The authors declare that the research was conducted in the absence of any commercial or financial relationships that could be construed as a potential conflict of interest.

## Publisher’s note

All claims expressed in this article are solely those of the authors and do not necessarily represent those of their affiliated organizations, or those of the publisher, the editors and the reviewers. Any product that may be evaluated in this article, or claim that may be made by its manufacturer, is not guaranteed or endorsed by the publisher.

## Supplementary material

The Supplementary material for this article can be found online at: https://www.frontiersin.org/articles/10.3389/fmicb.2023.1171423/full#supplementary-material

## Footnotes

^1^ http://cpc2.cbi.pku.edu.cn

^2^ http://sfold.wadsworth.org/

^3^ http://www.dna.bio.keio.ac.jp/mxfold2/

^4^ https://bibiserv.cebitec.uni-bielefeld.de/pkiss?id=pkiss_view_webservive

^5^ https://bibiserv.cebitec.uni-bielefeld.de/rnashapes?id=rnashapes_view_Webservice

^6^ http://rna.tbi.univie.ac.at//cgi-bin/RNAWebSuite/RNAfold.cgi
