# Supplementary material for: A novel lncRNA DFRV plays a dual function in influenza A virus infection
Source: Front Microbiol. 2023 May 25;14:1171423. doi: 10.3389/fmicb.2023.1171423 (PMC10248499; doi:10.3389/fmicb.2023.1171423)
Supplement: Supplementary file 1 [file Data_Sheet_1.DOCX]

Supplementary Material

A novel lncRNA DFRV plays a dual function in influenza A virus infection

Keyu Wang^1,†^, Meiliang Gong^1,†^, Sumin Zhao^2,†^, Chengcai Lai^3^, Lingna Zhao^4^, Sijie Cheng^5^, Min Xia^6^, Yuru Li^1^, Kun Wang^1^, Heqiang Sun^1^, Pingjun Zhu^7^, Yu Zhou^1,*^, Qiangguo Ao^8,*^ and Xinli Deng^1,*^

^1^Department of Clinical Laboratory, National Clinical Research Center for Geriatric Diseases, The second medical center of Chinese PLA General Hospital, Beijing 100853, China;

^2^The PLA Rocket Force Characteristic Medical Center, Beijing, 100088, China;

^3^Department of Pharmaceutical Sciences, Beijing Institute of Radiation Medicine, Beijing, China;

^4^Beijing Key Laboratory for Immunological Research on Chronic Diseases, School of Medicine and Institute for Immunology, Tsinghua University, Beijing 100084, China;

^5^Center for Disease Prevention and Control of ChangDe City, Hunan Province 415000, China;

^6^Department of Vascular Cell Biology, Max Plank Institute for Molecular Biomedicine, Münster 48149, Germany;

^7^Department of Respiratory and Critical Care Medicine, National Clinical Research Center for Geriatric Diseases, The second medical center of Chinese PLA General Hospital, Beijing 100853, China;

^8^Department of Nephrology, National Clinical Research Center for Geriatric Diseases, The second medical center of Chinese PLA General Hospital, Beijing 100853, China;

^†^These authors contributed equally to this work.

*** Correspondence:** zhouy427@163.com(Y.Z.); aoqg301@126.com(Q.A.); bjdxl@163.com(X.D.)

## Supplementary Figures and Tables


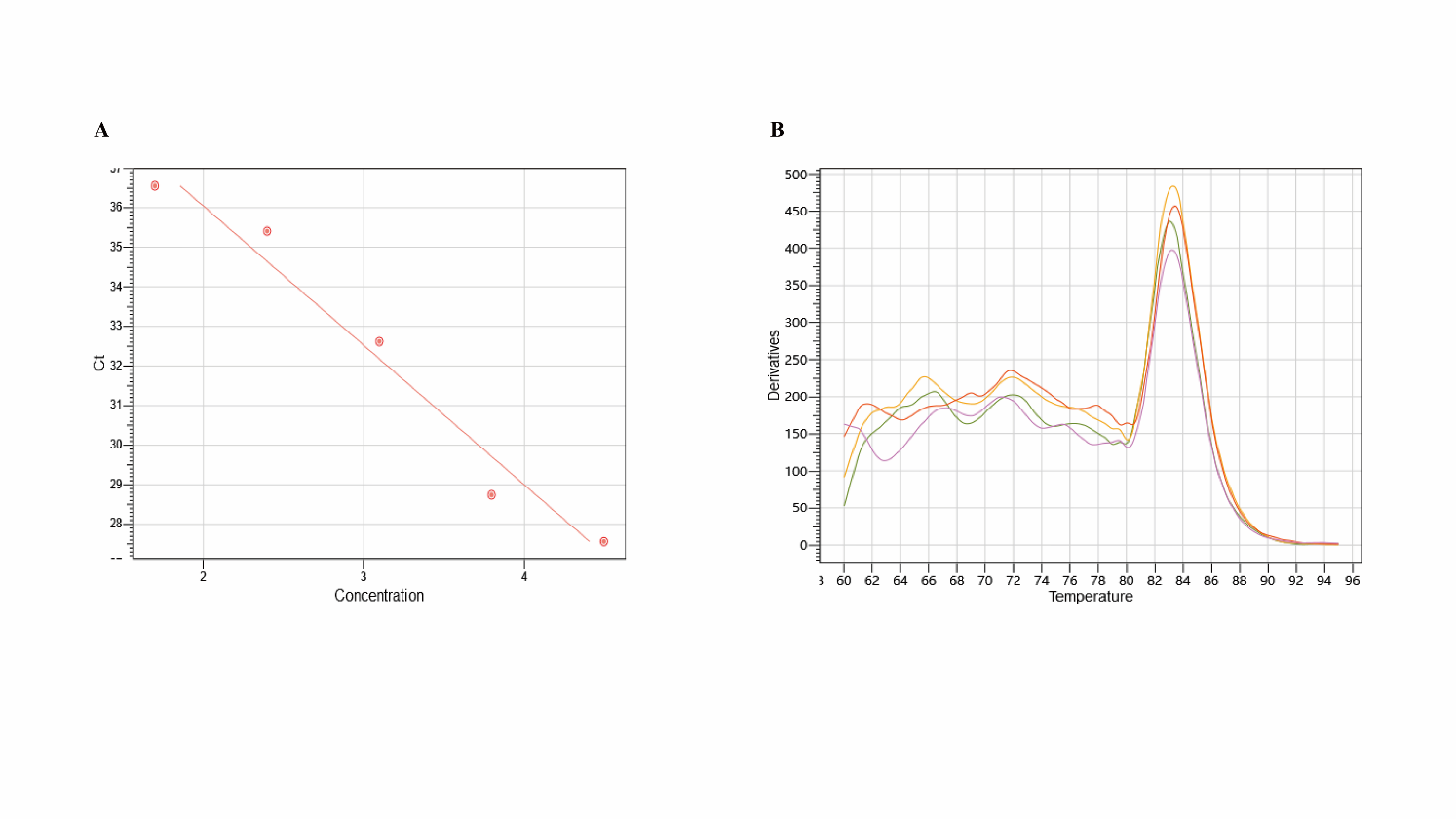


**Supplementary Figure 1. The primer efficiency curves and the melting curves for DFRV.**

**(A)** The primer efficiency curves for DFRV. **(B)** The melting curves for DFRV.


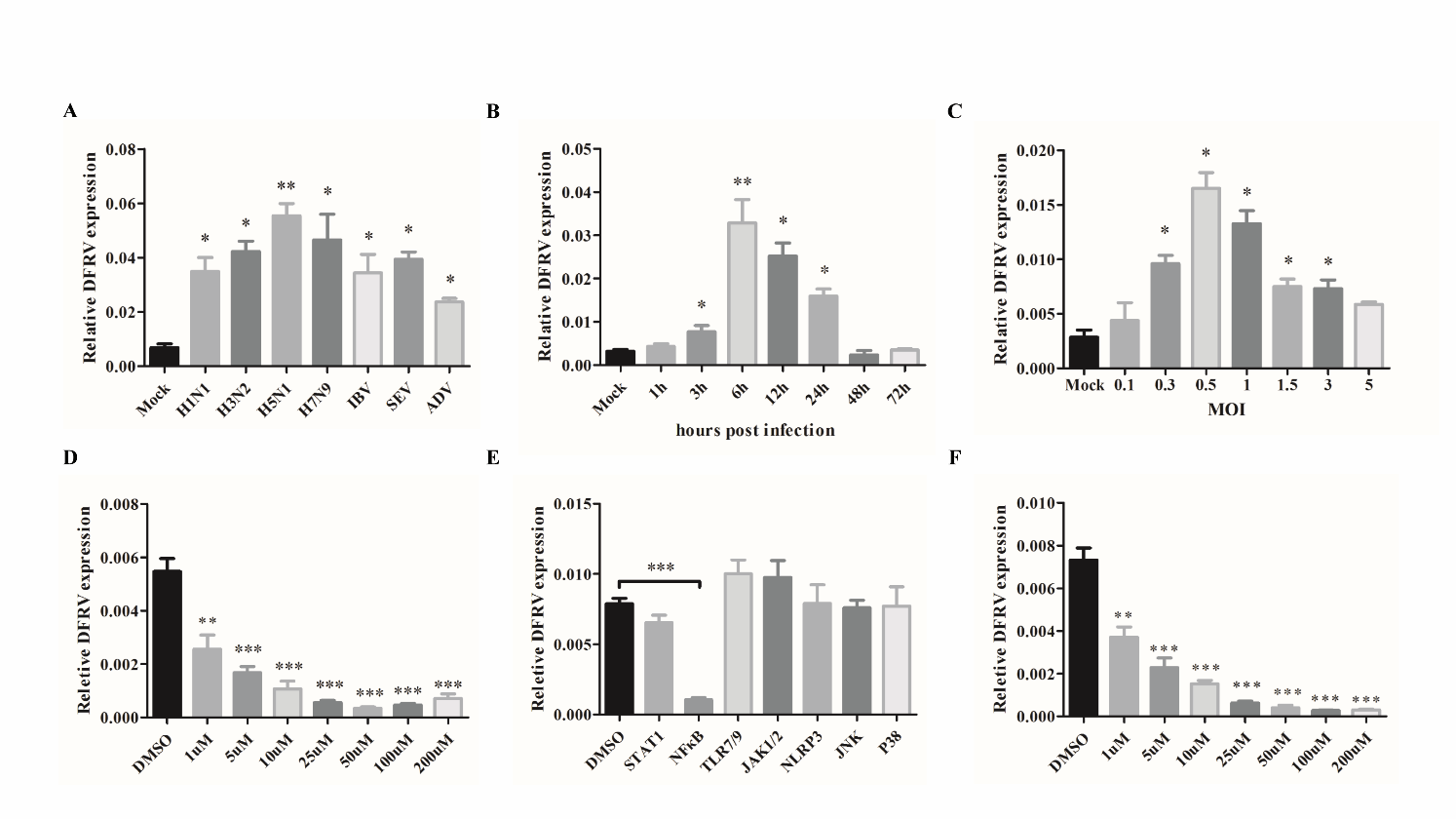


**Supplementary Figure 2. lncRNA DFRV is significantly elevated in IAV infection and dependent on NFκB signaling pathway. (A)** THP-1 cells were infected with H1N1, H3N2, H5N1, H7N9, influenza B virus (IBV), Sendai virus (SEV) and Adenovirus (ADV) at MOI=1 for 24h, qRT-PCR was performed to determine the DFRV expression. **(B)** THP-1 cells were infected with BJ501 (MOI=1) for different hours post infection, and **(C)** THP-1 cells were infected with BJ501 at different multiplicity of infection (MOI) for 24h, qRT-PCR was performed to determine the DFRV expression. **(D)** A549 cells were pretreated with NFκB signaling pathway inhibitor in different concentrations followed by BJ501 infection for 24 h. qRT-PCR was performed to determine the DFRV expression. **(E)** THP-1 cells were pretreated with 7 signaling pathway inhibitors followed by BJ501 infection for 24 h. qRT-PCR was performed to determine the DFRV expression. **(F)** THP-1 cells were pretreated with NFκB signaling pathway inhibitor in different concentrations followed by BJ501 infection for 24 h. qRT-PCR was performed to determine the DFRV expression. All of the data are shown as the mean ± SD; n = 3. *P < 0.05; **P < 0.01;***P < 0.001.


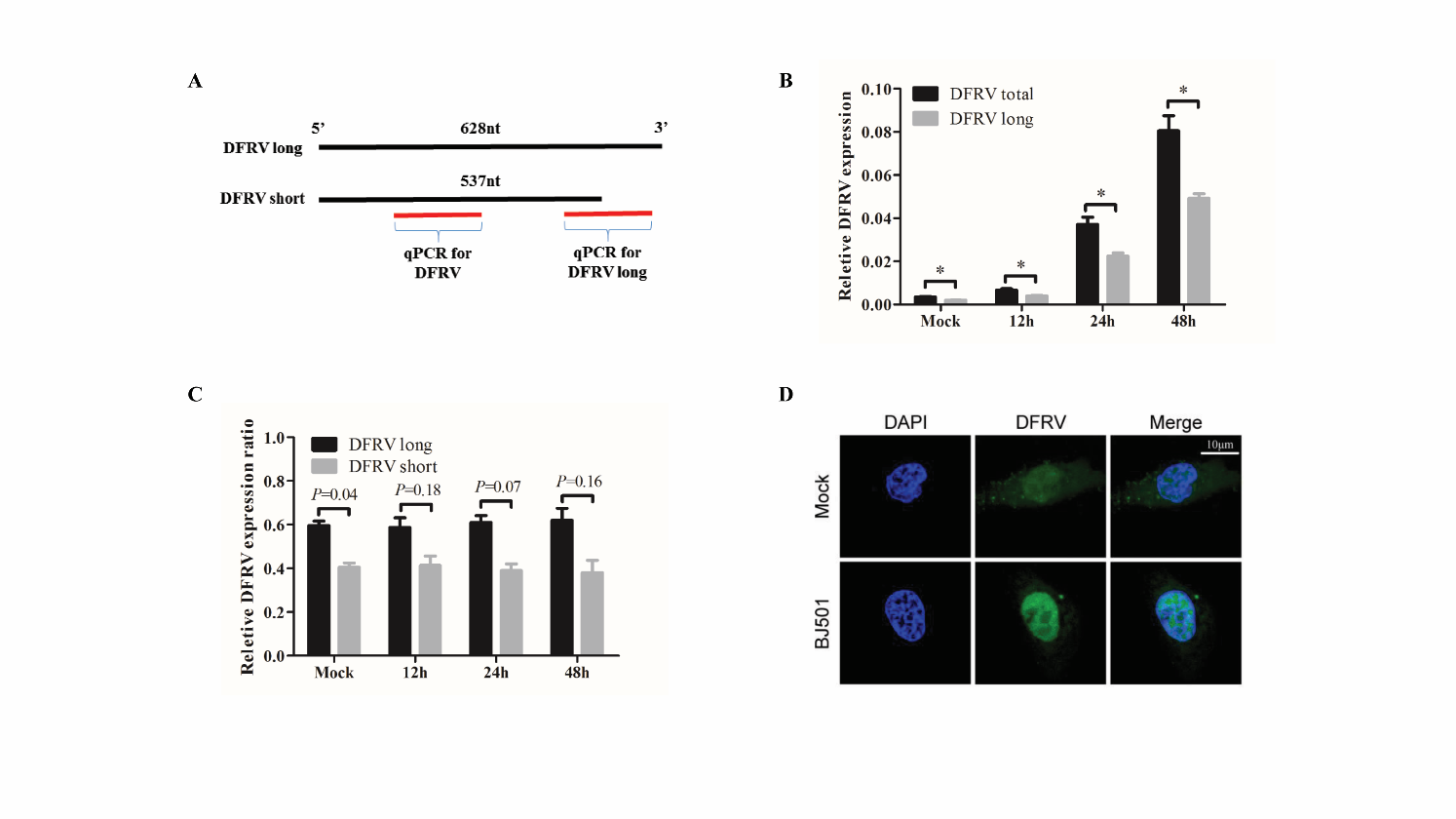


**Supplementary Figure 3. Identification and characterization of DFRV. (A)** Schematic of the primers for qRT-PCR targeting DFRV. **(B)** A549 cells were infected with BJ501 (MOI=1), and qRT-PCR was performed to determine the expression level of DFRV long and DFRV total. **(C)** The expression ratio of DFRV long and short was calculated. **(D)** DFRV intracellular localization visualized by RNA-FISH in A549 cells stimulated with MOCK or BJ501 (MOI=1). DFRV was labeled with green fluorescence, and the nuclei were stained by DAPI (blue). Scale bar: 10μm.


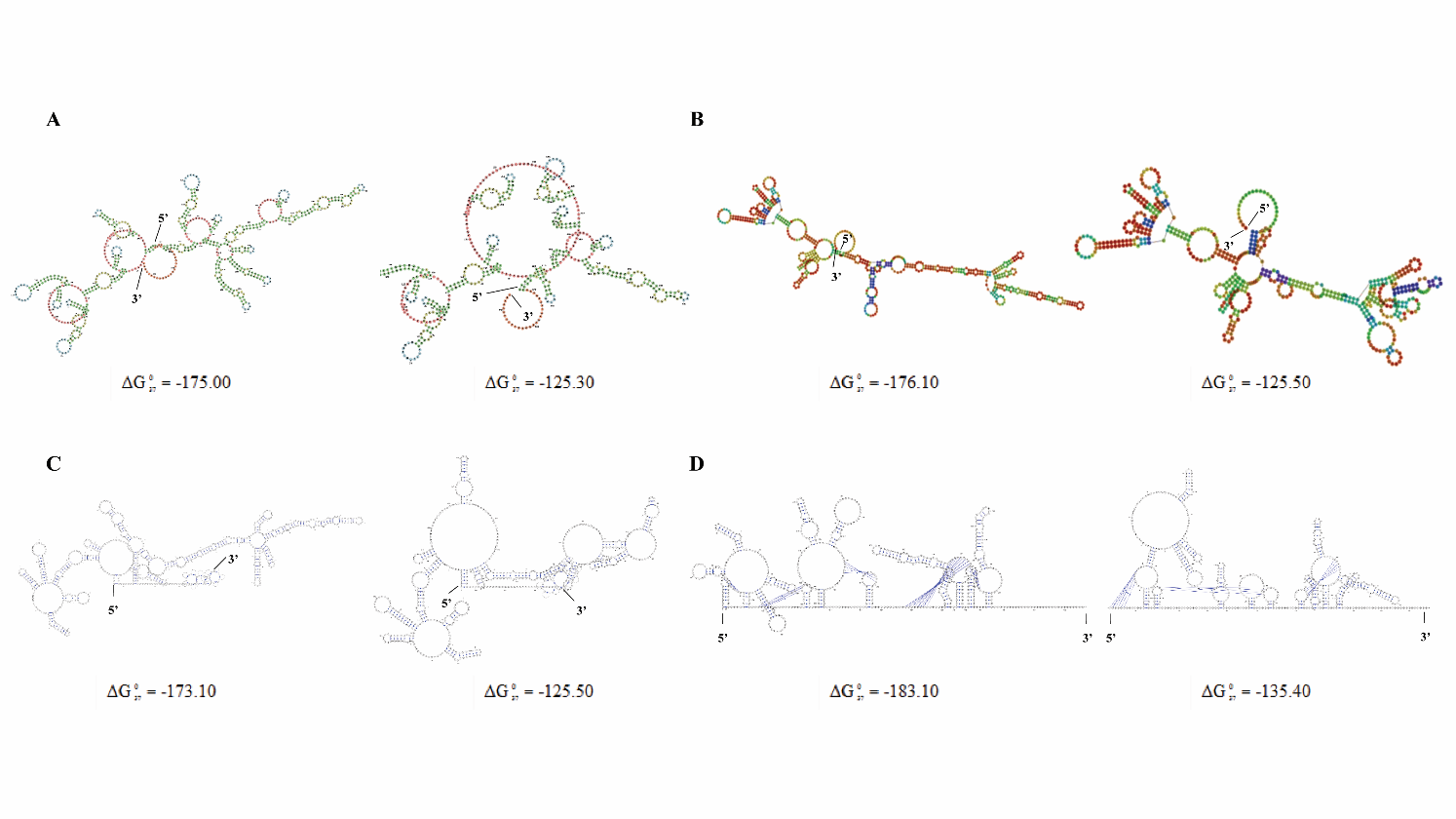


**Supplementary Figure 4. The secondary structure prediction of DFRV long and short.** Secondary structure predictions of DFRV were performed through mxfold **(A)**, RNAfold **(B)**, RNAshapes **(C)** and pKiss **(D)**, and the minimum free energy (MFE) of DFRV long (left) and short (right) were exhibited.

**
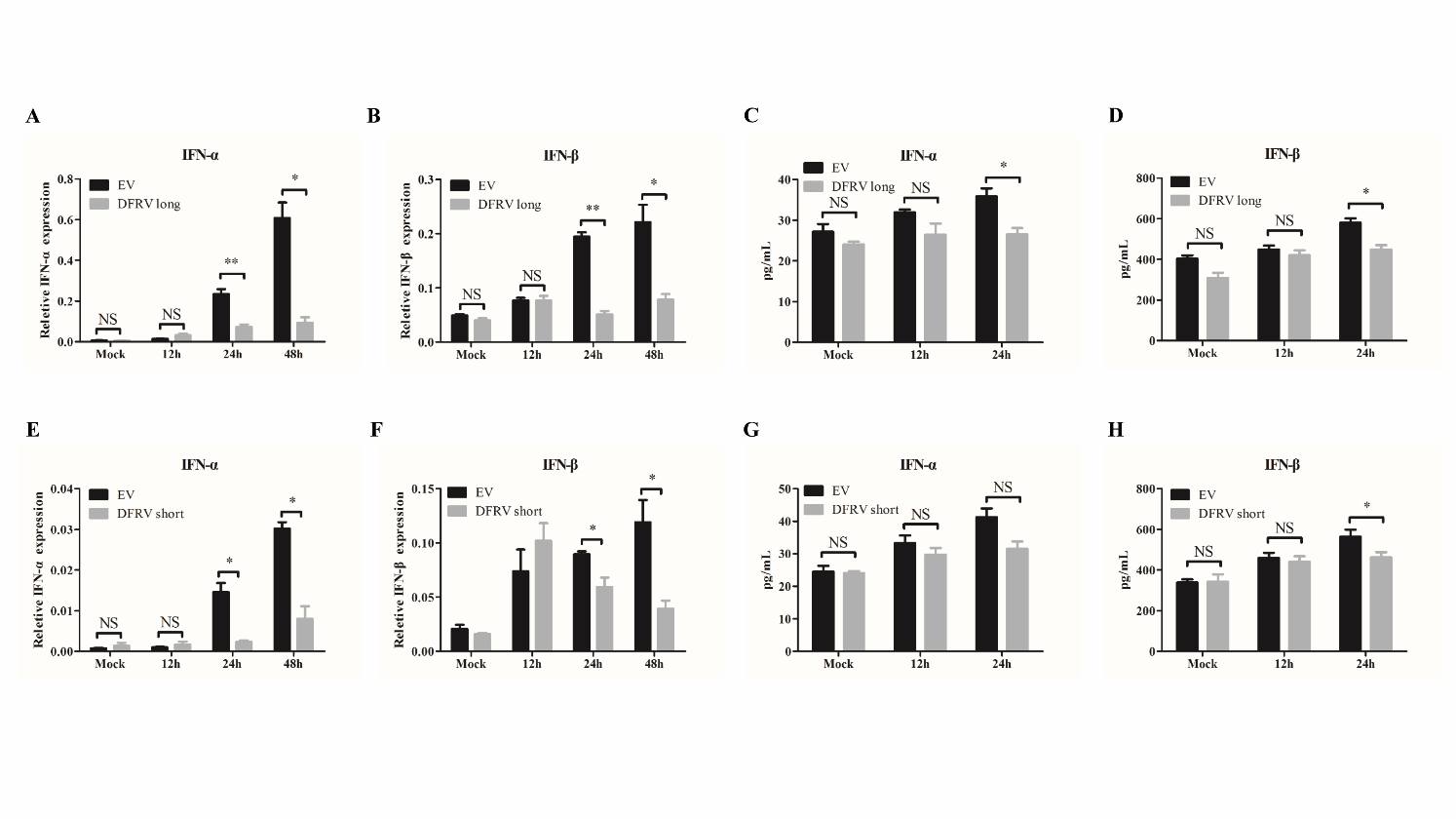
**

**Supplementary Figure 5. DFRV play an important role in antiviral immune response.** DFRV long or short were transiently overexpressed in A549 cells for transfected with plasmids for 14h, and infected with BJ501 (MOI=1) in 12h, 24h and 48h. The mRNA expression level of IFN-α **(A, E)** and IFN-β **(B, F)** was determined by qRT-PCR. A549 culture supernatants were collected to measure the IFN-α **(C, G)** and IFN-β **(D, H)** expression by ELISA. All of the data are shown as the mean ± SD; n = 3. *P < 0.05; **P < 0.01;***P < 0.001, EV: Empty vector.


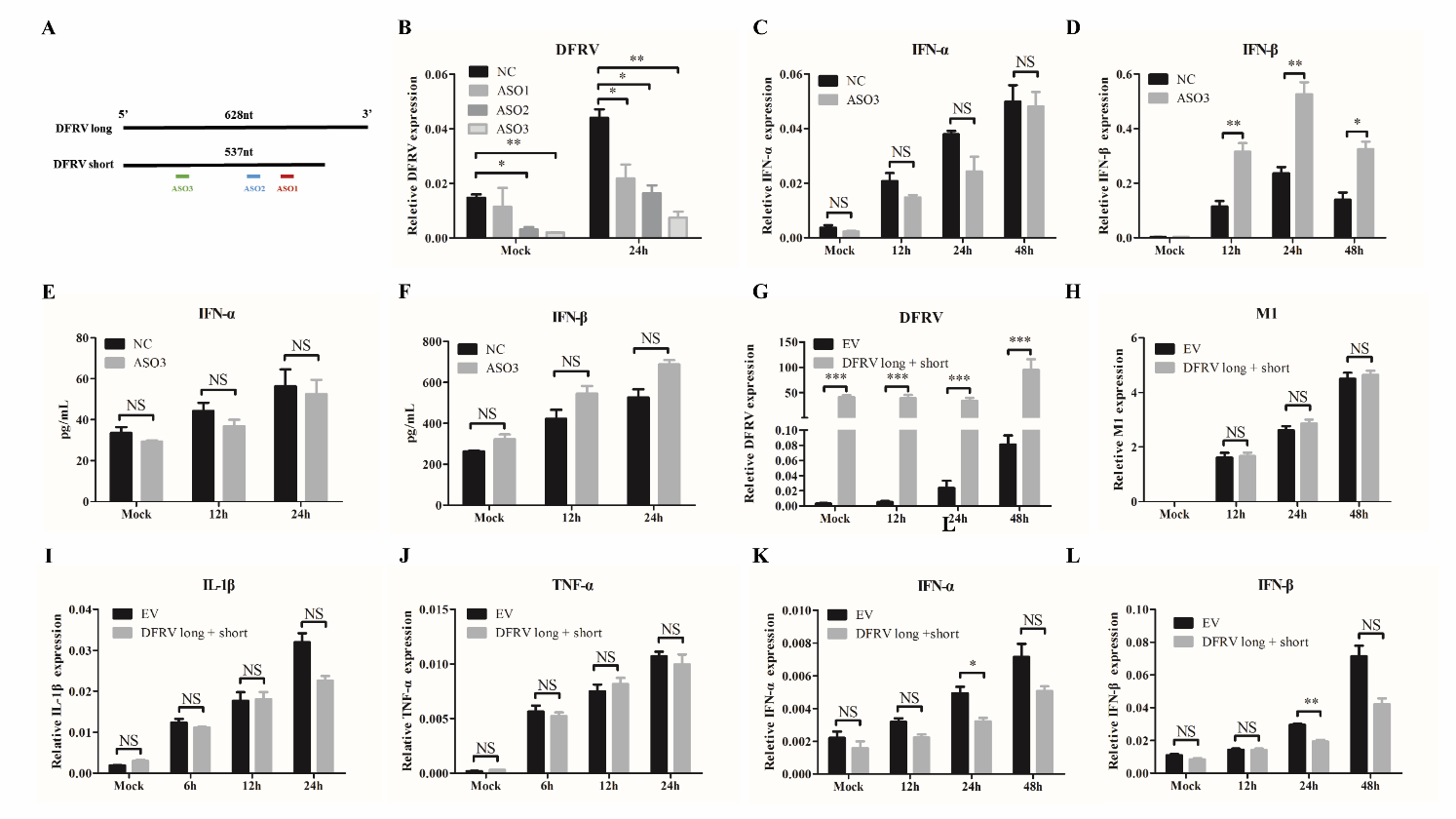


**Supplementary Figure 6. The strategy of knock down the expression of DFRV. (A)** Schematic of the ASOs targeting DFRV. **(B)** A549 cells were transfected with ASOs for 14h, and DFRV expression were determined by qRT-PCR. The mRNA expression level of IFN-α **(C)** and IFN-β **(D)** was determined by qRT-PCR. A549 culture supernatants were collected to measure the IFN-α **(E)** and IFN-β **(F)** expression by ELISA. **(G-L)** A549 cells were co-transfected with DFRV long and short plasmids for 14h, and infected with BJ501 (MOI=1) in 12h, 24h and 48h. the mRNA expression level of DFRV **(G)**, influenza M1 **(H)**, IL-1β **(I)**, TNF-α **(J)**, IFN-α **(K)**, IFN-β **(L)** were determined by qRT-PCR. All of the data are shown as the mean ± SD; n = 3. *P < 0.05; **P < 0.01;***P < 0.001, EV: Empty vector.

| **Supplementary Table 1. Primer pairs used for qRT-PCR.** | | |
| --- | --- | --- |
| Gene | Forward | Reverse |
| DFRV | 5’-CCCTCTCCGCACTAACATCT-3’ | 5’-GTAGGCACCAGCGAGACAAC-3’ |
| DFRV-long | 5’-GGAGCCGCTCTAGGCTTTTG-3’ | 5’-CACCCTTCGACCCCGGAA-3’ |
| PCBP2 | 5’-TGCCATTCCACAGCCAGATT-3’ | 5’-TCCAAACCTGCCCAATAGCC-3’ |
| U6 | 5’-CTCGCTTCGGCAGCACA-3’ | 5’-AACGCTTCACGAATTTGCGT-3’ |
| GAPDH | 5’-GGTGGTCTCCTCTGACTTCAACA-3’ | 5’-GTTGCTGTAGCCAAATTCGTTGT-3’ |
| M1 | 5’-AAGACCAATCCTGTCACCTCTG-3’ | 5’-CAAAACGTCTACGCTGCAGTCC-3’ |
| IFN-α | 5’- GTGAGGAAATACTTCCAAAGA-3’ | 5’-TCTCATGATTTCTGCTCTGACA-3’ |
| IFN-β | 5’-AGCTGAAGCAGTTCCAGAAG-3’ | 5’-AGTCTCATTCCAGCCAGTGC-3’ |
| IL-1β | 5’-ACAGTGGCAATGAGGATGAC-3’ | 5’-CTGAAGCCCTTGCTGTAGTG-3’ |
| TNF-α | 5’-CAGAGGGCCTGTACCTCATC-3’ | 5’-GGAAGACCCCTCCCAGATAG-3’ |

| **Supplementary Table 2: The sequence of DFRV long and shorts.** | |
| --- | --- |
| DFRV | 5'ATTTCTTTCATTCCTTCTAGATTTATTACATGGAACTCTTTAAAAAAGAGCTTTCCCTTATCAATTACAGATGAACTATATACAGTTCTTCCTAAAAGGAAAGTTAAATGCTTAATTCTTTCCTTTTAAGAGTAAGGAGTTTATACAATGGTCACCTCTAACGTGGCAAGTTAGTTTTTTTCCTTTTCTTTTTTGAGTATCCCTGTAGATTACTATCTTCATCCTTTCTCTTTCCACCCATCCCTTTTTCCTCCTCTCCACTTCTTGATGCATGGCGACTGCTCCCTCTCCGCACTAACATCTGAACTTTCCAGCCAGTCCACTAGGGGCCACTGGTTTCCTTTCTGCCCCCACGCTCCCTCTCTATCGCAATTCGTCGCTTGCTAGCAGCCGTTGTCTCGCTGGTGCCTACGCTGTCAAAGGAGCCGCTCTAGGCTTTTGGGAGGCCGTCTGCATTGCGT3' |
| DFRV long | 5'GGGTTGCAAAATGGTGACTTTTCAACTCTCATTCCTTTTATTTCTTTCATTCCTTCTAGATTTATTACATGGAACTCTTTAAAAAAGAGCTTTCCCTTATCAATTACAGATGAACTATATACAGTTCTTCCTAAAAGGAAAGTTAAATGCTTAATTCTTTCCTTTTAAGAGTAAGGAGTTTATACAATGGTCACCTCTAACGTGGCAAGTTAGTTTTTTTCCTTTTCTTTTTTGAGTATCCCTGTAGATTACTATCTTCATCCTTTCTCTTTCCACCCATCCCTTTTTCCTCCTCTCCACTTCTTGATGCATGGCGACTGCTCCCTCTCCGCACTAACATCTGAACTTTCCAGCCAGTCCACTAGGGGCCACTGGTTTCCTTTCTGCCCCCACGCTCCCTCTCTATCGCAATTCGTCGCTTGCTAGCAGCCGTTGTCTCGCTGGTGCCTACGCTGTCAAAGGAGCCGCTCTAGGCTTTTGGGAGGCCGTCTGCATTGCGTGGGGAAGGCTTGGACGACGCAATCGCCTCGCCGGCCCCCGGGGTGGTTAGCGCTTCCGGGGTCGAAGGGTGCGCGGGGTTGAAAGCAGGCCGCTCCGCCCCGTCCCCCTCCCAGACCAGCAGAGGCAGAAAAAAAAAAAAAAAAAAAAAAAAAAA3’ |
| DFRV short | 5'GGGTTGCAAAATGGTGACTTTTCAACTCTCATTCCTTTTATTTCTTTCATTCCTTCTAGATTTATTACATGGAACTCTTTAAAAAAGAGCTTTCCCTTATCAATTACAGATGAACTATATACAGTTCTTCCTAAAAGGAAAGTTAAATGCTTAATTCTTTCCTTTTAAGAGTAAGGAGTTTATACAATGGTCACCTCTAACGTGGCAAGTTAGTTTTTTTCCTTTTCTTTTTTGAGTATCCCTGTAGATTACTATCTTCATCCTTTCTCTTTCCACCCATCCCTTTTTCCTCCTCTCCACTTCTTGATGCATGGCGACTGCTCCCTCTCCGCACTAACATCTGAACTTTCCAGCCAGTCCACTAGGGGCCACTGGTTTCCTTTCTGCCCCCACGCTCCCTCTCTATCGCAATTCGTCGCTTGCTAGCAGCCGTTGTCTCGCTGGTGCCTACGCTGTCAAAGGAGCCGCTCTAGGCTTTTGGGAGGCCGTCTGCATTGCGTGGGGAAGGCTTGGACGACGCAATCGCCTCGCCGGCCCAAAAAAAAAAAAAAAAAAAAAAAAAAAA3’ |
