# Supplementary figures and images for: A novel lncRNA DFRV plays a dual function in influenza A virus infection
Source: Front Microbiol. 2023 May 25;14:1171423. doi: 10.3389/fmicb.2023.1171423 (PMC10248499; doi:10.3389/fmicb.2023.1171423)

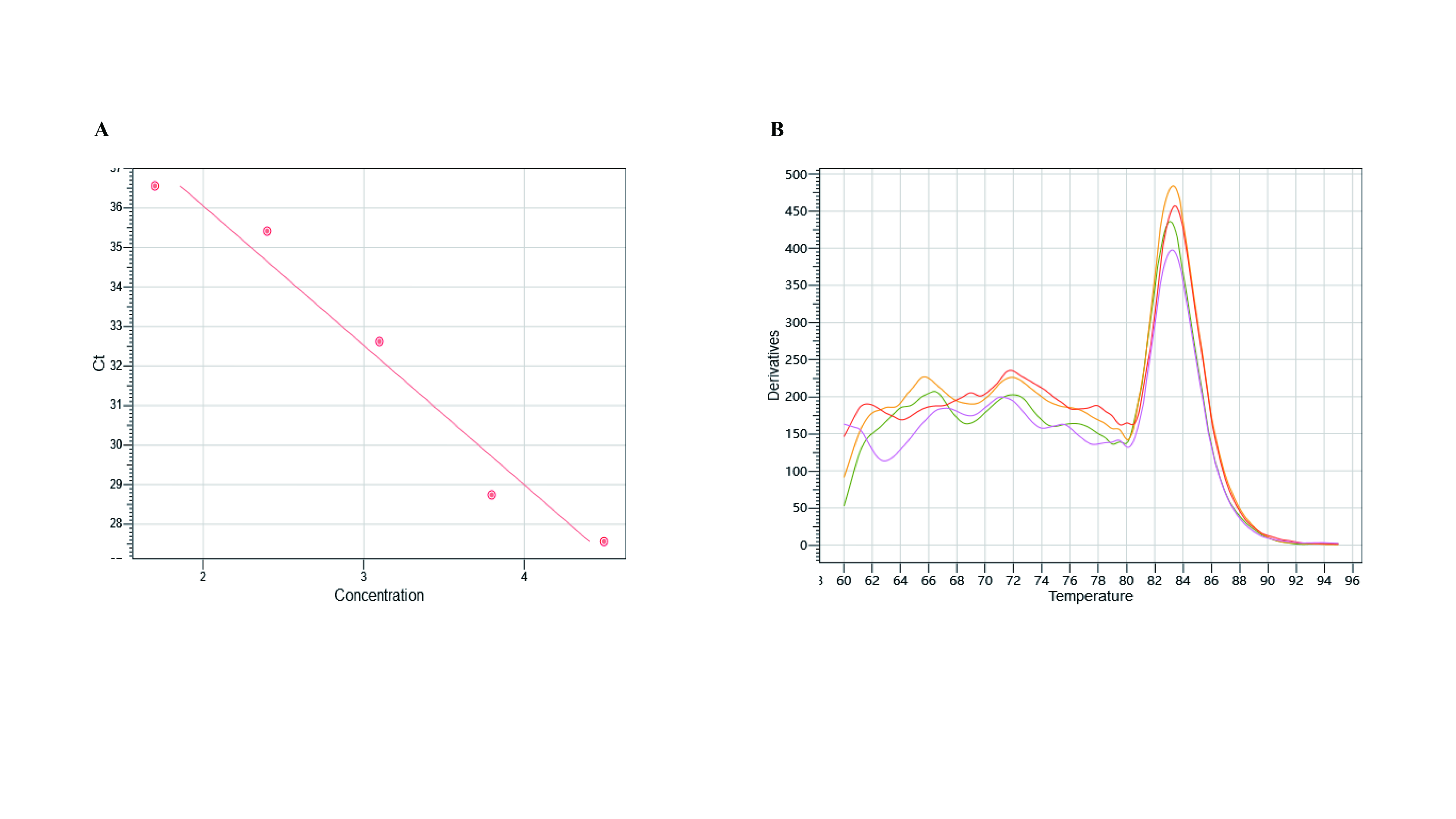

Supplement: Supplementary file 2 [file Data_Sheet_2.ZIP › Supplementary Material/Figure S1.tif]

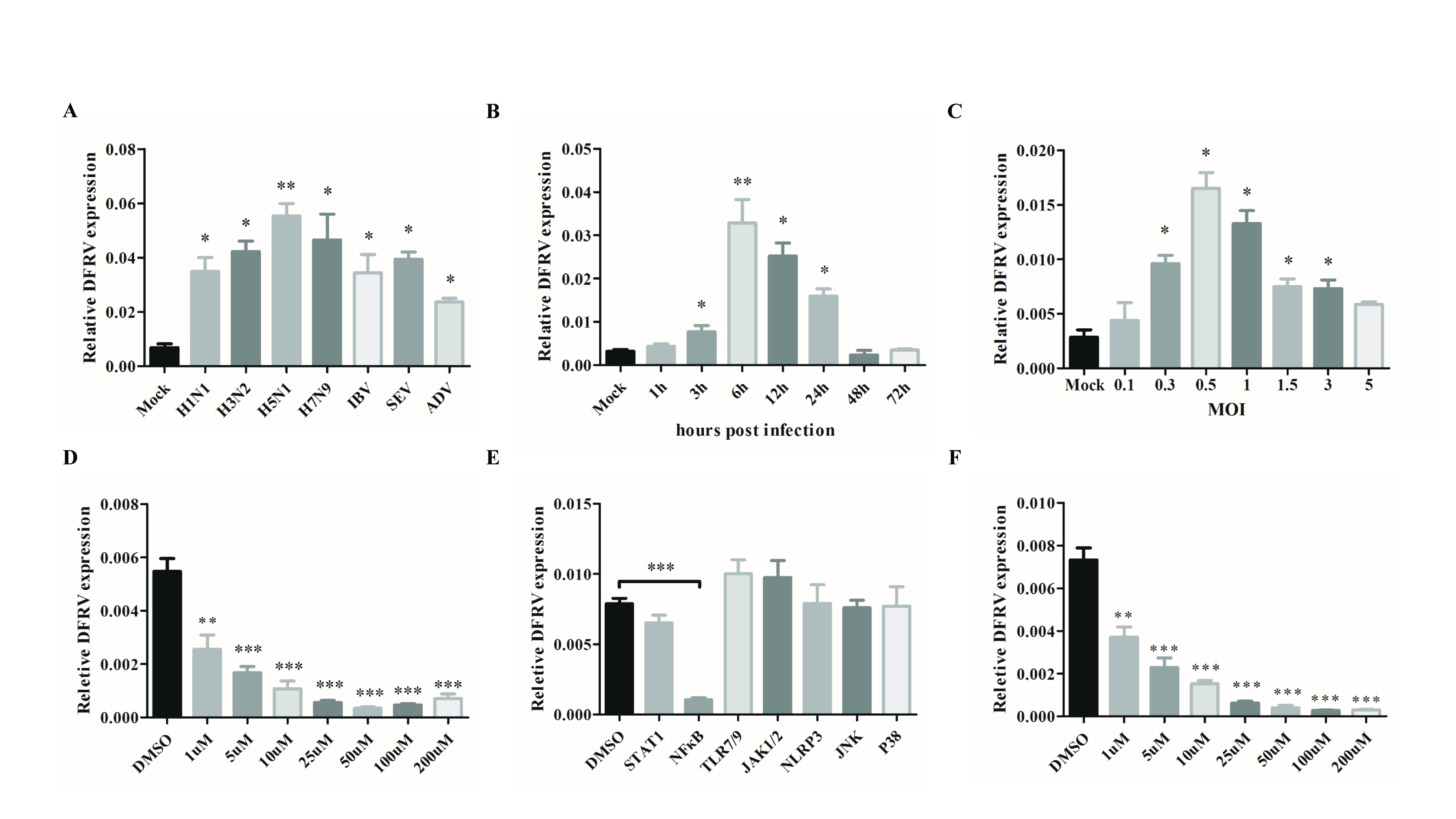

Supplement: Supplementary file 2 [file Data_Sheet_2.ZIP › Supplementary Material/FigureS2.tif]

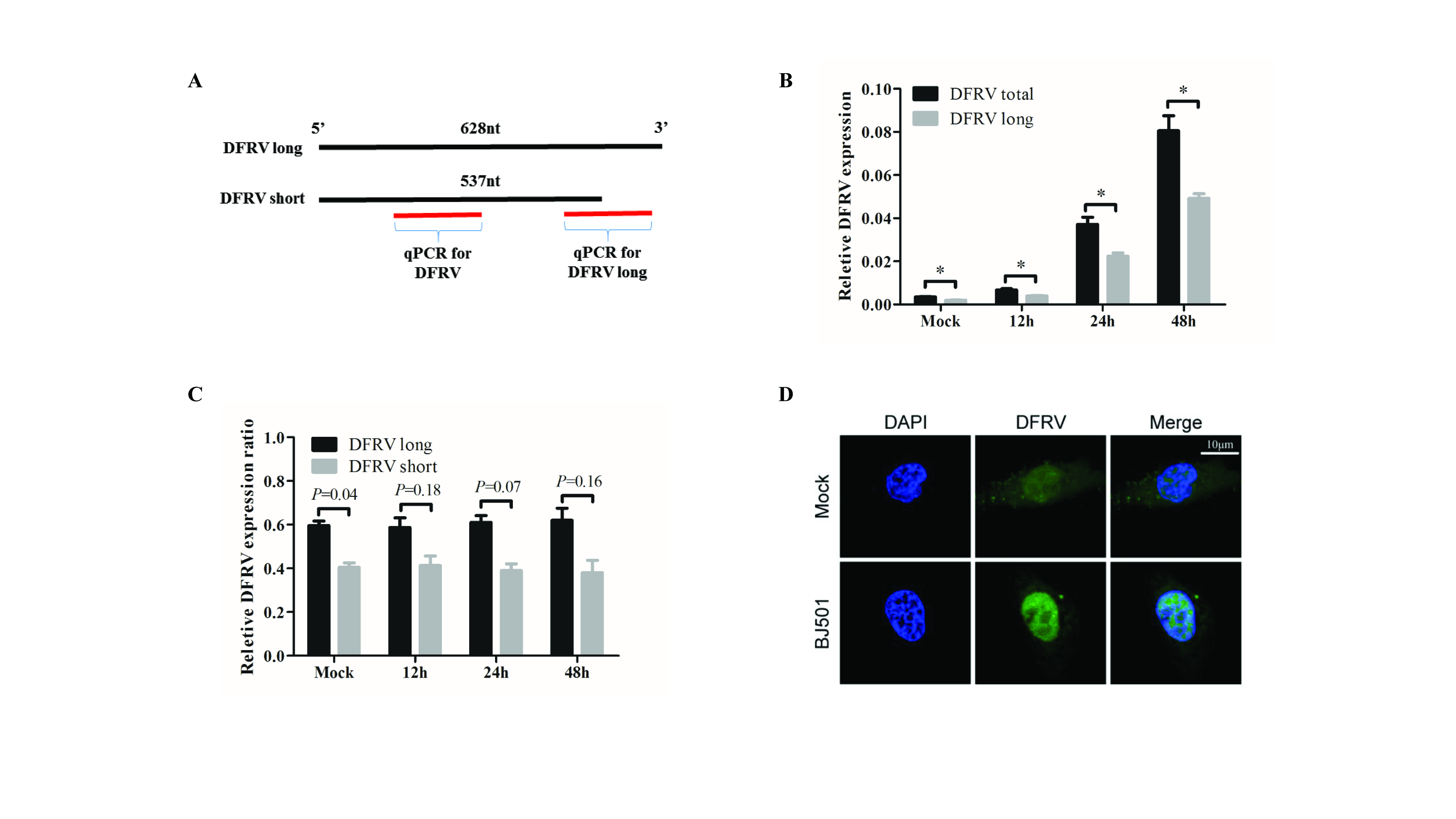

Supplement: Supplementary file 2 [file Data_Sheet_2.ZIP › Supplementary Material/FigureS3.tif]

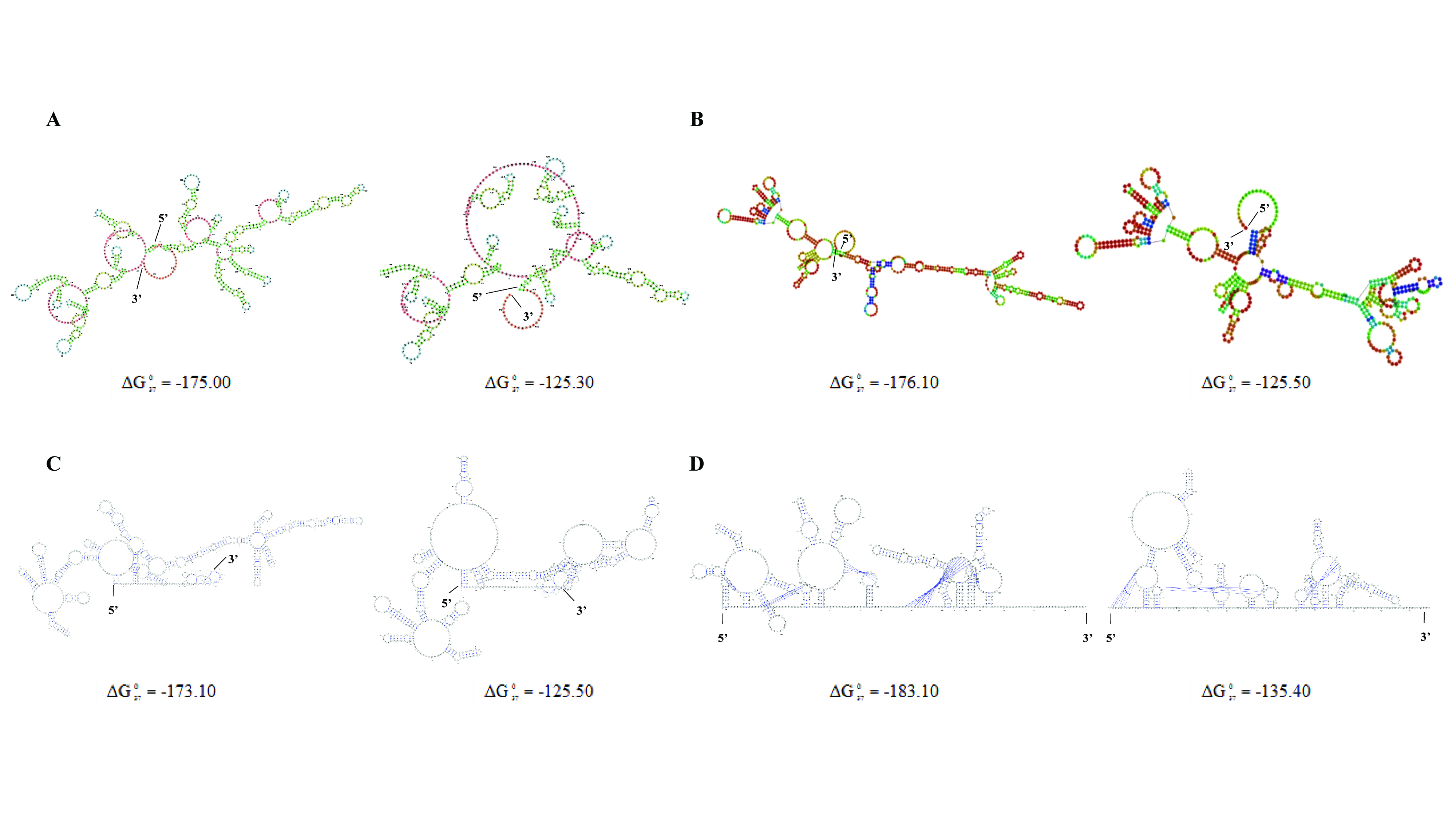

Supplement: Supplementary file 2 [file Data_Sheet_2.ZIP › Supplementary Material/FigureS4.tif]

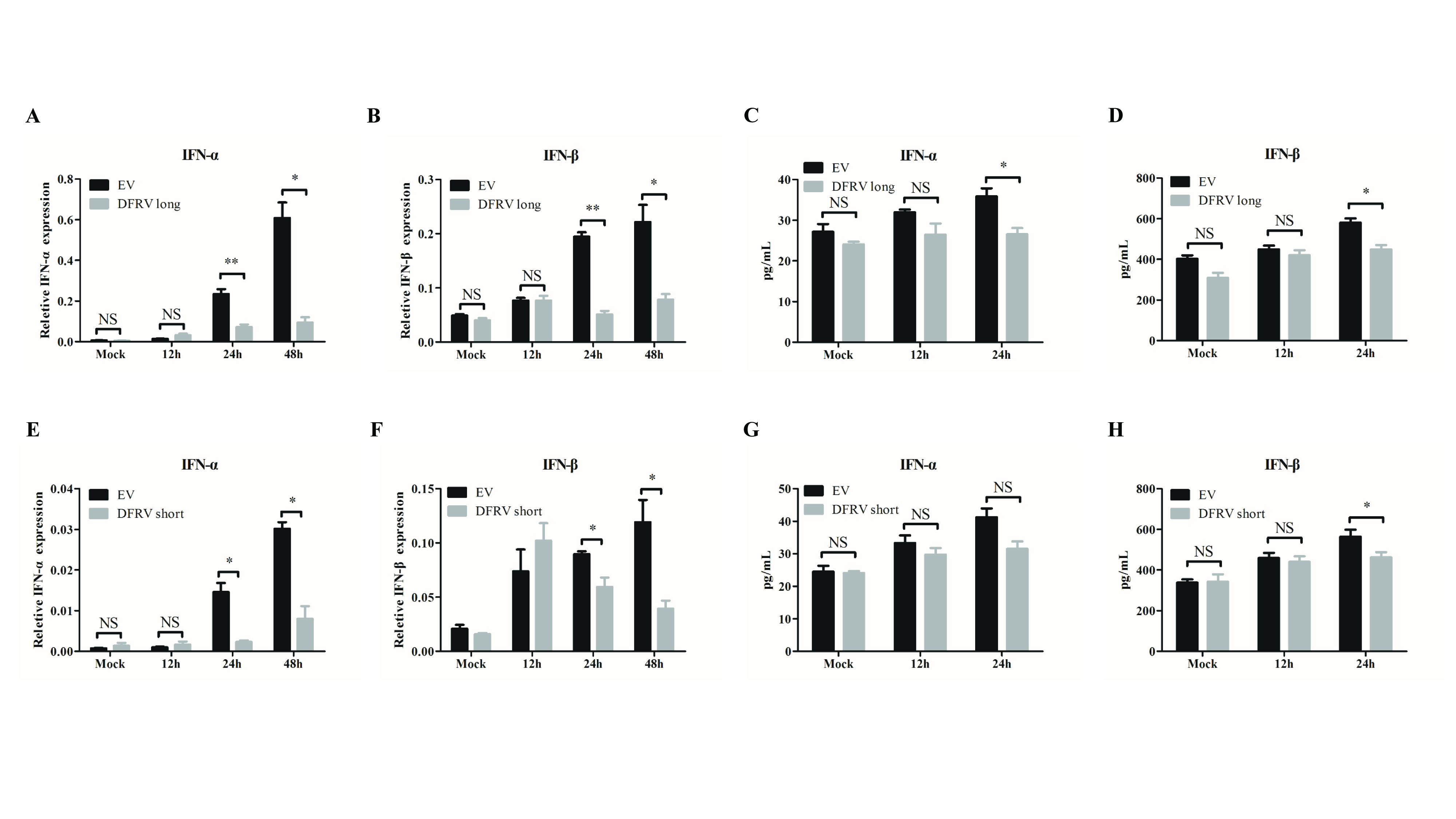

Supplement: Supplementary file 2 [file Data_Sheet_2.ZIP › Supplementary Material/FigureS5.tif]

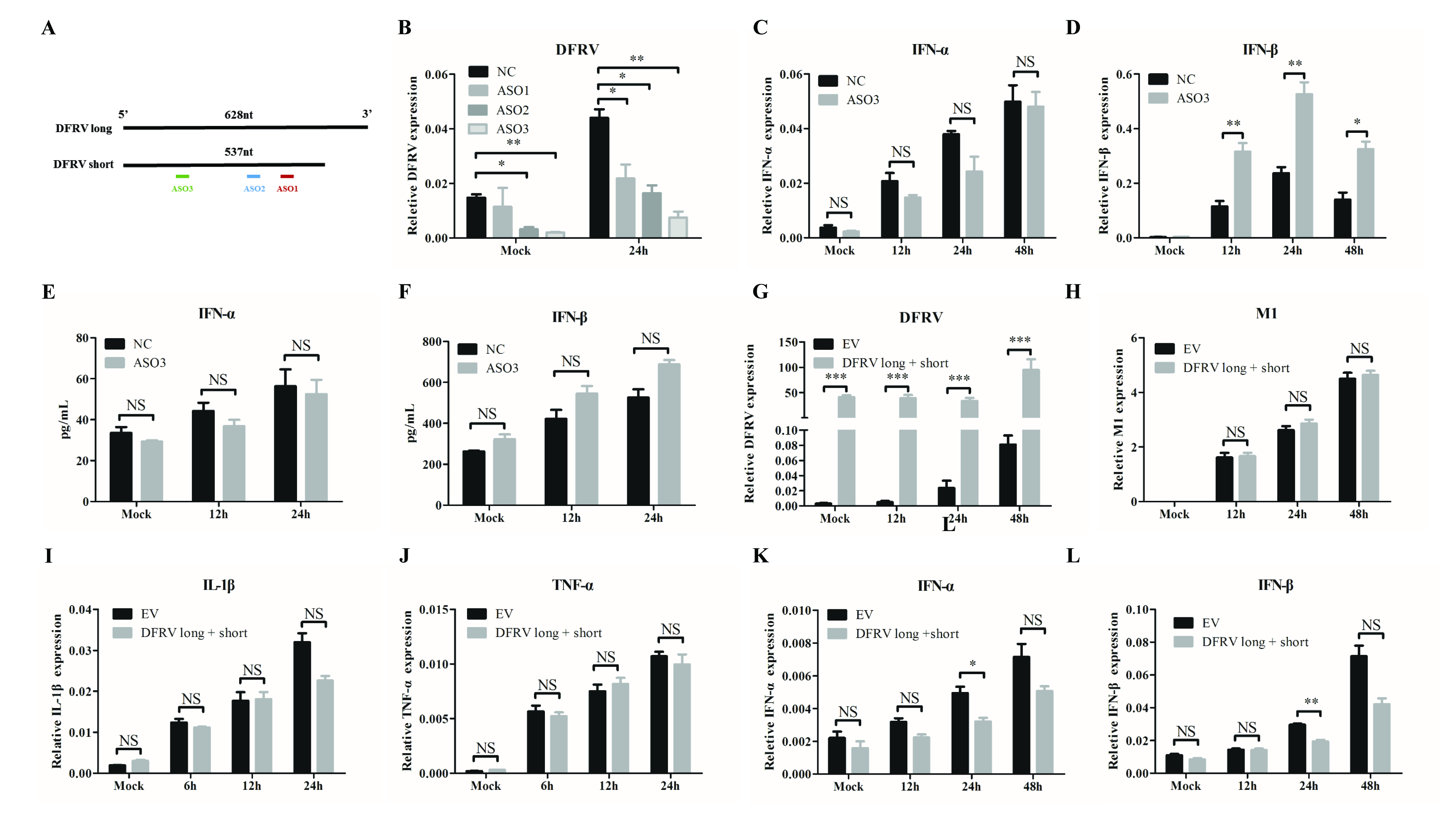

Supplement: Supplementary file 2 [file Data_Sheet_2.ZIP › Supplementary Material/FigureS6.tif]

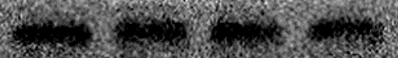

Supplement: Supplementary file 3 [file Data_Sheet_3.ZIP › Western Blot-revised/AKT.tif]

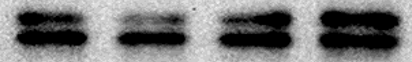

Supplement: Supplementary file 3 [file Data_Sheet_3.ZIP › Western Blot-revised/ERK.tif]

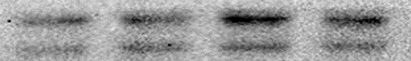

Supplement: Supplementary file 3 [file Data_Sheet_3.ZIP › Western Blot-revised/JNK.tif]

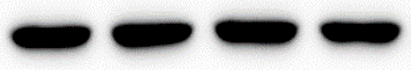

Supplement: Supplementary file 3 [file Data_Sheet_3.ZIP › Western Blot-revised/NFKB.tif]

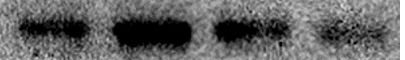

Supplement: Supplementary file 3 [file Data_Sheet_3.ZIP › Western Blot-revised/P-AKT.tif]

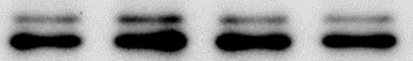

Supplement: Supplementary file 3 [file Data_Sheet_3.ZIP › Western Blot-revised/P-ERK.tif]

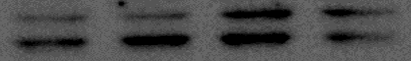

Supplement: Supplementary file 3 [file Data_Sheet_3.ZIP › Western Blot-revised/P-JNK.tif]

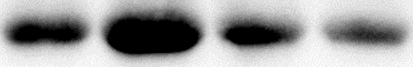

Supplement: Supplementary file 3 [file Data_Sheet_3.ZIP › Western Blot-revised/P-NFKB.tif]

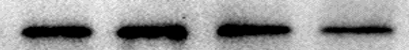

Supplement: Supplementary file 3 [file Data_Sheet_3.ZIP › Western Blot-revised/P-P38.tif]

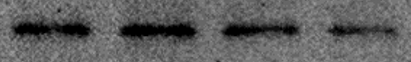

Supplement: Supplementary file 3 [file Data_Sheet_3.ZIP › Western Blot-revised/P-PI3K.tif]

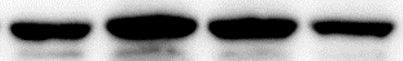

Supplement: Supplementary file 3 [file Data_Sheet_3.ZIP › Western Blot-revised/P-STAT3.tif]

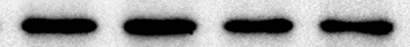

Supplement: Supplementary file 3 [file Data_Sheet_3.ZIP › Western Blot-revised/P38.tif]

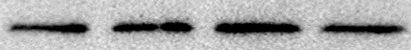

Supplement: Supplementary file 3 [file Data_Sheet_3.ZIP › Western Blot-revised/PI3K.tif]

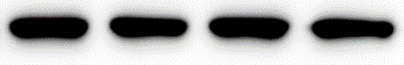

Supplement: Supplementary file 3 [file Data_Sheet_3.ZIP › Western Blot-revised/STAT3.tif]

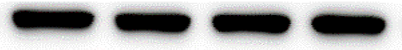

Supplement: Supplementary file 3 [file Data_Sheet_3.ZIP › Western Blot-revised/β-actin.tif]
